# Blp1 protein shows virulence-associated features and elicits protective immunity to *Acinetobacter baumannii* infection

**DOI:** 10.1186/s12866-019-1615-3

**Published:** 2019-11-21

**Authors:** Jūratė Skerniškytė, Emilija Karazijaitė, Julien Deschamps, Renatas Krasauskas, Julija Armalytė, Romain Briandet, Edita Sužiedėlienė

**Affiliations:** 1 Institute of Biosciences, Life Sciences Center, Vilnius University, Saulėtekio ave. 7, LT-10257 Vilnius, Lithuania; 2 Micalis Institute, INRA, AgroParisTech, Université Paris-Saclay, 78350 Jouy-en-Josas, France

**Keywords:** *Acinetobacter baumannii*, Blp1, Virulence, Biofilm, Vaccine

## Abstract

**Background:**

Multidrug resistant *Acinetobacter baumannii* is one of the major infection agents causing nosocomial pneumonia. Therefore, new therapeutic approaches against this bacterium are needed. Surface-exposed proteins from bacterial pathogens are implicated in a variety of virulence-related traits and are considered as promising candidates for vaccine development.

**Results:**

We show in this study that a large Blp1 protein from opportunistic pathogen *A. baumannii* is encoded in all examined clinical strains of globally spread international clonal lineages I (IC I) and II (IC II). The two *blp1* gene variants exhibit lineage-specific distribution profile. By characterization of *blp1* deletion mutants and their complementation with *blp1* alleles we show that *blp1* gene is required for *A. baumannii* biofilm formation and adhesion to epithelial cells in IC I strain but not in the IC II strain. Nevertheless both alleles are functional in restoring the deficient phenotypes of IC I strain. Moreover, the *blp1* gene is required for the establishing of *A. baumannii* virulence phenotype in nematode and murine infection models. Additionally, we demonstrate that C-terminal 711 amino acid fragment of Blp1 elicits an efficient protection to lethal *A. baumannii* infection in a murine model using active and passive immunization approaches. Antiserum obtained against Blp1-specific antigen provides opsonophagocytic killing of *A. baumannii* in vitro.

**Conclusions:**

Lineage-specific variants of surface-exposed components of bacterial pathogens complicate the development of new therapeutic approaches. Though we demonstrated different impact of Blp1 variants on adherence of IC I and IC II strains, Blp1-specific antiserum neutralized *A. baumannii* strains of both clonal lineages. Together with the observed increased survival rate in vaccinated mice these results indicate that *A. baumannii* Blp1 protein could be considered as a new vaccine candidate.

## Background

*Acinetobacter baumannii* is a widely spread opportunistic pathogen causing pneumonia, sepsis and soft tissue infections [1]. *A. baumannii* is the most dangerous to critically ill patients and is responsible for hospital infection outbreaks, especially in the intensive care units [2, 3]. Antibiotic therapy is complicated due to the global spread of multidrug-resistant (MDR) strains, non-susceptible to most antibiotic classes [4]. Therefore, *A. baumannii* has been considered as a top priority pathogen for which new drugs and therapeutic options are urgently needed [5].

Among strategies considered, the vaccine-based approaches show the greatest potential [6]. However, the main challenge to identify an efficient vaccine candidate against *A. baumannii* infections still remains [6]. The desirable antigens should be highly prevalent surface-exposed bacterial proteins and possess high degree of conservation among clinical strains. However, the vast majority of clinical *A. baumannii* isolates possess a thick outer layer of polysaccharides (capsule), which efficiently protects pathogens from the host immunity by shielding cell surface antigens [7, 8]. To be able to adhere to the host cells and form biofilms, capsulated pathogens use long extended molecules such as adhesins or pili-like structures, penetrating the thick capsule [9, 10]. Exploring the characteristics of such surface-exposed proteins and their role in pathogenesis might reveal the new targets suitable for antimicrobial therapy against clinical *A. baumannii* MDR strains.

The *A. baumannii* Blp1 protein (also named as BapA, GenBank accession number: WP_126655828) has been recently identified to exhibit structural organization, similar to that of a giant *A. baumannii* protein called Bap (biofilm-associated protein) [11]. Blp1 exhibits a tripartite structure, which includes C-terminal and N-terminal domains, separated by a large portion of a repetitive region containing combinations of various motifs including bacterial Ig-like (Big) domains. The Big-like domains, present in both Blp1 and Bap proteins, are a characteristic feature of some bacterial fimbrial and non-fimbrial adhesins, which enhance the attachment to various materials, such as host cells or abiotic surfaces [9, 12–14]. Indeed, it has been demonstrated recently that the deletion of *blp1* gene decreases *A. baumannii* biofilm formation and adhesion to the lung epithelial cells A549 [11]. Unlike the *bap* gene, which seems to be truncated in a number of *A. baumannii* genomes, the intact *blp1* allele was common in *A. baumannii* genomes analyzed [11]. Therefore, the predicted bacterial surface-associated features of Blp1 protein and the prevalence of *blp1* gene in bacterial genomes make Blp1 an attractive antigen candidate for vaccination-based strategies against MDR *A. baumannii*.

Two major *A. baumannii* pandemic clonal lineages, namely IC I and IC II, cause the highest percentage of hospital acquired *A. baumannii* infections worldwide [2, 3]. Genome analysis of the strains belonging to these two clonal lineages demonstrated the significant differences in genetic determinants of antimicrobial resistance [15–17]. Due to high carbapenem-resistance, the IC II lineage strains are characterized through the increased nosocomial spread in many countries during the recent years [2, 3]. Since lineage-depended genetic differences were also observed in surface-related components [15–17], the substantial role of these specific genes variants in *A. baumannii* dissemination and pathogenesis could be assumed. The objective of this study was to investigate the incidence and distribution of *blp1* gene variants among clinical *A. baumannii* strains, belonging to the most globally spread clonal lineages, to assess the role of *blp1* gene in virulence-related features and, most importantly, to evaluate the efficiency of Blp1 protein-based vaccine against *A. baumannii* in murine infection model.

## Results

### The distribution of *blp1* alleles in clinical *A.baumannii* strains of international clonal lineages I and II

**Fig. 1:**
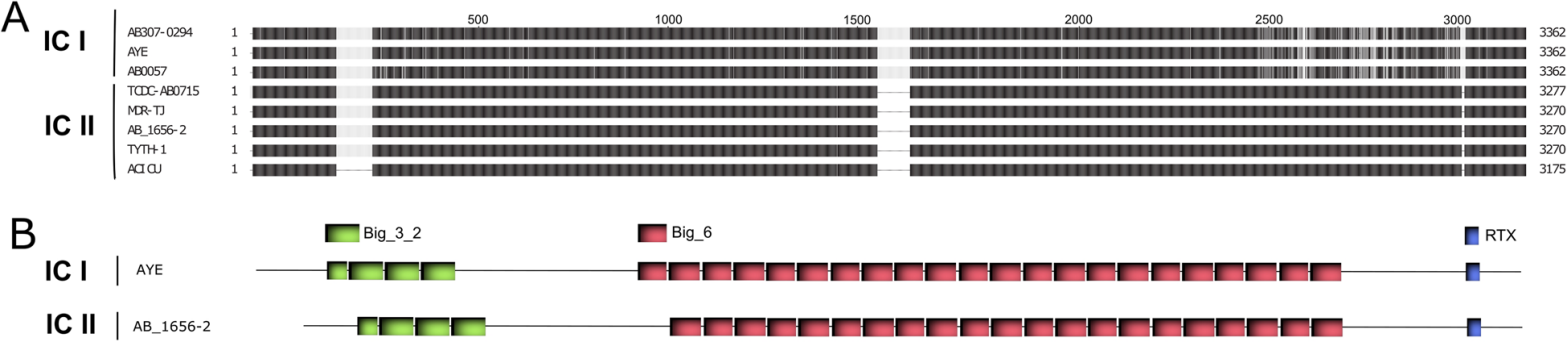
Allelic variations of Blp1 among the strains of two major *A. baumannii* pandemic clones. **a** – an alignment of Blp1 sequences in *A. baumannii* IC I and IC II strains, available in NCBI Genbank; black areas indicate 100% identity, variable regions are in grey and the absence of the region is indicated by the thin strip; numbers demonstrate the position of amino acids; alignment was prepared using TexShade tool. **b** – organization of Blp1 protein domains in representative strains of IC I (AYE) and IC II (AB_1656–2) clonal lineages*.*; green and red boxes represent bacterial immunoglobulin-like 3_2 type (Big_3_2), and immunoglobulin-like 6 type (Big_6) domains, respectively, blue boxes represent RTX domains; domain organization was identified using HHMER tool

*A. baumannii* is characterized by a high degree of genomic variability [15–17] and this trait was recently demonstrated for *blp1* gene [11]. Therefore, we were interested in the prevalence and variability of *blp1* gene in the clinically relevant strains belonging to two predominant clones IC I and IC II. First, we have performed analysis of the genomes of strains with the assigned clonal lineage I or II, available in NCBI Genbank and have observed a clear association of two different *blp1* allele variants with either IC I or IC II lineage strains (Fig. 1a). The number of bacterial Ig-like repeat units (Bigs) encoded in *blp1* gene varied among the strains, typically 26 units found in gene variant in IC I strains and 24–25 units in its counterpart in IC II strains (Fig. 1b). Two types of repeats, named Big_3_2 and Big_6, were identified in both alleles. The Blp1 protein variants encoded in the genomes of IC I and IC II strains showed 71–74% identity. Approximately 160 amino acids at the Blp1 C-terminus harboring RTX-toxin domain and T1SS signal sequence were conserved in both protein variants. Whereas the region of approximately 540 amino acids between Big repeats and RTX-domain, contained IC-specific variations (Fig. 1a). The observed *A. baumannii* Blp1 C-terminal sequence variations, commonly found in bacterial surface exposed structures [18, 19], are in line with the proposed Blp1 surface association.

Next, to confirm the relationship of *blp1* gene alleles with the IC lineages, we designed the primer pairs (Additional file 1: Table S1) specifically targeting each allele variant and screened for their prevalence in the collection of clinical *A. baumannii* isolates representing IC I (*n* = 72) and IC II (*n* = 50) clonal lineages. All tested IC I strains were found to possess *blp1*_IC I_ allele, while all IC II strains contained *blp1*_IC II_ allele, showing the distribution profile, strictly specific for clonal lineage, thereby confirming the trait observed from the in silico analysis (Fig. 1a).

### The loss of *blp1* gene differently impacts the ability of *A*. *baumannii* IC I and IC II strains to form biofilm

The observed *blp1* polymorphism among IC I and IC II strains prompted us to investigate their functionality differences by assessing the virulence-related *A. baumannii* properties. For this purpose, two representative clinical *A. baumannii* isolates belonging to IC I and IC II clonal lineages, designated Ab_IC I_ and Ab_IC II_, respectively, were selected from the collection of previously characterized clinical isolates [20] and used for further analysis. Their characteristics are represented in Additional file 1: Table S1. The Ab_IC I_ and Ab_IC II_ strains were assigned to ST231 and ST208 sequence types, respectively, according to the Oxford MLST typing scheme. Both these sequence types were found to be globally disseminated [21, 22]. Markerless deletions of *blp1* allelic variants in Ab_IC I_ and Ab_IC II_ strains were generated as described in Materials and Methods. Notably, the *blp1* deletions did not result in any alterations of the growth of mutant strains (data not shown). For the complementation experiments, plasmids p*blp1*_IC I_ and p*blp1*_IC II_ carrying *blp1* allelic variants were constructed and introduced into Ab_IC I_Δ*blp1* and Ab_IC II_Δ*blp1* strains as described in Materials and Methods. The loss of endogenous *blp1* gene expression in the Δ*blp1* mutants and restoration of *blp1* expression by complementation, has been verified by qPCR using primers BaprB/BapRaiR (Additional file 1: Table S1; Additional file 2: Figure S1).

**Fig. 2:**
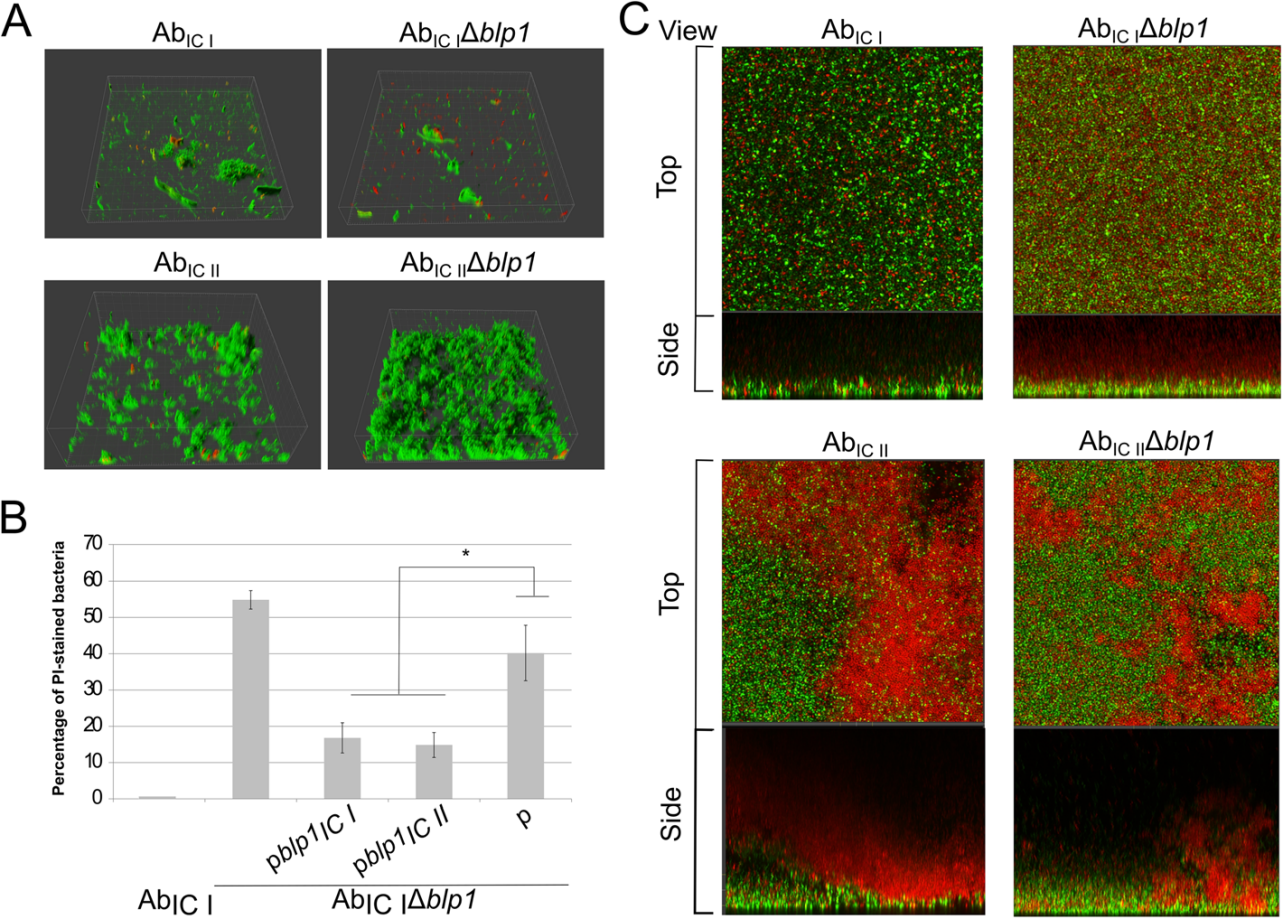
CLSM analysis of biofilms formed by the *A. baumannii* IC I and IC II strains. **a** – 3D visualization of initial attachment to the plastic by *A. baumannii blp1* gene deletion mutants and parental strains assessed after 2 h of incubation; bacteria were stained with SYTO9 (green) and propidium iodide (red). **b** – number of propidium iodide stained bacteria after 2 h of incubation, compared to the total amount of cells and expressed as a percentage; error bars represent standard errors from the measurements of six different CLSM pictures, significance was assessed by *t*-test, (*P < 0.05). **c** – mature biofilm formation after 24 h of incubation; *A. baumannii* parental strains and *blp1* gene deletion mutants were stained with SYTO9 (green) and propidium iodide (red); representative top and side views are given

Firstly, the biofilm formation of Ab_IC I_Δ*blp1* and Ab_IC II_Δ*blp1* mutants and their *blp1*-complemented strains was assessed. Bacteria were grown on the plastic surface and the formed structures were analyzed by confocal laser scanning microscopy (CLSM) using SYTO9 and propidium iodide (PI) staining as described in Materials and Methods. Interestingly, 2 h post-seeding, the initial attachment of bacteria to the plastic surface, based on the increase of PI-stained (red stain) versus total amount of bacteria (green stain) was affected in Ab_IC I_Δ*blp1* mutant, demonstrating up to 55% increase in PI-stained bacteria compared with the parental strain (Fig. 2a, upper panels). Contrary to the Ab_IC I_Δ*blp1* mutant, the Ab_IC II_Δ*blp1* strain showed no alterations in the initial attachment compared to the parent, evident through the lack of PI-stained bacteria and through the formation of more uniform biofilm structure similar to parental strain (Fig. 2a, lower panels). To verify that the observed phenotype of Ab_IC I_Δ*blp1* was indeed *blp1-*dependent, we introduced plasmids p*blp1*_IC I_ and p*blp1*_IC II_ carrying respective *blp1* alleles into Ab_IC I_Δ*blp1* bacteria and assessed their attachment to the plastic surface. Notably, both *blp1* alleles efficiently restored the phenotype of Ab_IC I_Δ*blp1* mutant manifested by the reduction in PI-stained cells compared to the Ab_IC I_Δ*blp1* mutant carrying control plasmid (Fig. 2b and Additional file 3: Figure S2).

Next, we analyzed changes in the structure of mature biofilms formed by Ab_IC I_Δ*blp1* and Ab_IC II_Δ*blp1* strains and their parental strains after growth for 24 h. The *blp1*_IC I_ gene deletion resulted in the reduced thickness of the layer of viable cells in a mature biofilm formed by IC I strain and in an increased amount of PI-stained layer, which most likely represents the extracellular DNA (eDNA) producing cells [23] (Fig. 2c, upper panels). Notably, the structure of a mature biofilm formed by Ab_IC II_ strain differed from that produced by Ab_IC I_ strain, evident through a non-uniform appearance of PI-stained zones, although no apparent differences in the biofilm structure were observed between Ab_IC II_Δ*blp1* strain and its parent (Fig. 2c, lower panels). Clearly visible upper PI-stained layer in Ab_IC II_ biofilm, which was absent in Ab_IC I_, indicates the different biofilm maturation compared to Ab_IC I_ strain.

We have noticed that Ab_IC II_ strain tended to rapidly form multi-layered clusters in 2 h after seeding (Fig. 2a). Further maturation of these clusters accumulating a high amount of eDNA, possibly could provide an efficient protection against environmental stress, as this behavior was described for other pathogenic bacteria [24]. Therefore, the results, presented above suggest, that *blp1* allele variants have distinct impact on the biofilm formation by *A. baumannii* IC I and IC II strains.

### The *blp1* gene is required for the adhesion of Ab_IC I_ strain to lung epithelium cells

**Fig. 3:**
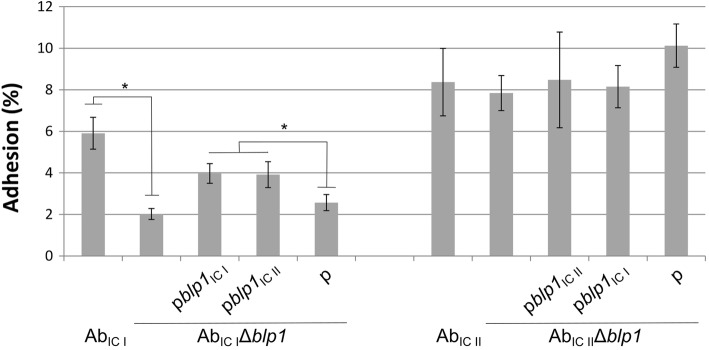
Adhesion of *A. baumannii* IC I and IC II strains to the lung epithelium LL/2 cells. Bacterial adhesion to the LL/2 cells after 1.5 h of incubation was expressed as a percentage of the CFUs of adhered bacteria compared to the total number of CFUs of the initial inoculum; error bars represent standard errors from at least three independent experiments; significance was assessed by *t*-test, (*P < 0.05)

To investigate the role of IC-associated *blp1* variants on the adhesion phenotype, strains comprising Ab_IC I_ and Ab_IC II_, their *blp1* deletion mutants and *Δblp1* strains complemented with plasmids containing respective *blp1* allelic variants were tested for the adhesive properties using mouse lung epithelium LL/2 cells (see Materials and Methods). The *blp1* deletion resulted in a reduction of adhesion of Ab_IC I_Δ*blp1* strain by three-fold (Fig. 3, left panel), whereas the loss of *blp1* allele in IC II strain had no effect on the adhesion (Fig. 3, right panel). Nevertheless, while dispensable for the adhesion of Ab_IC II_ strain, the *blp1*_IC II_ allele was able to restore adhesive properties of Ab_IC I_Δ*blp1* mutant at the level, similar to that of cognate version of *blp1* thereby indicating functionality of both *blp1* variants (Fig. 3, left panel).

Therefore, both *blp1* gene variants encode proteins that are functional in supporting adhesion of *A. baumannii* to lung epithelium cells, though *blp1* deletion in Ab_IC II_ strain had no impact on this phenotype.

### Loss of *blp1* gene affects *A*. *baumannii* virulence in *Caenorhabditis elegans* and murine infection models

**Fig. 4:**
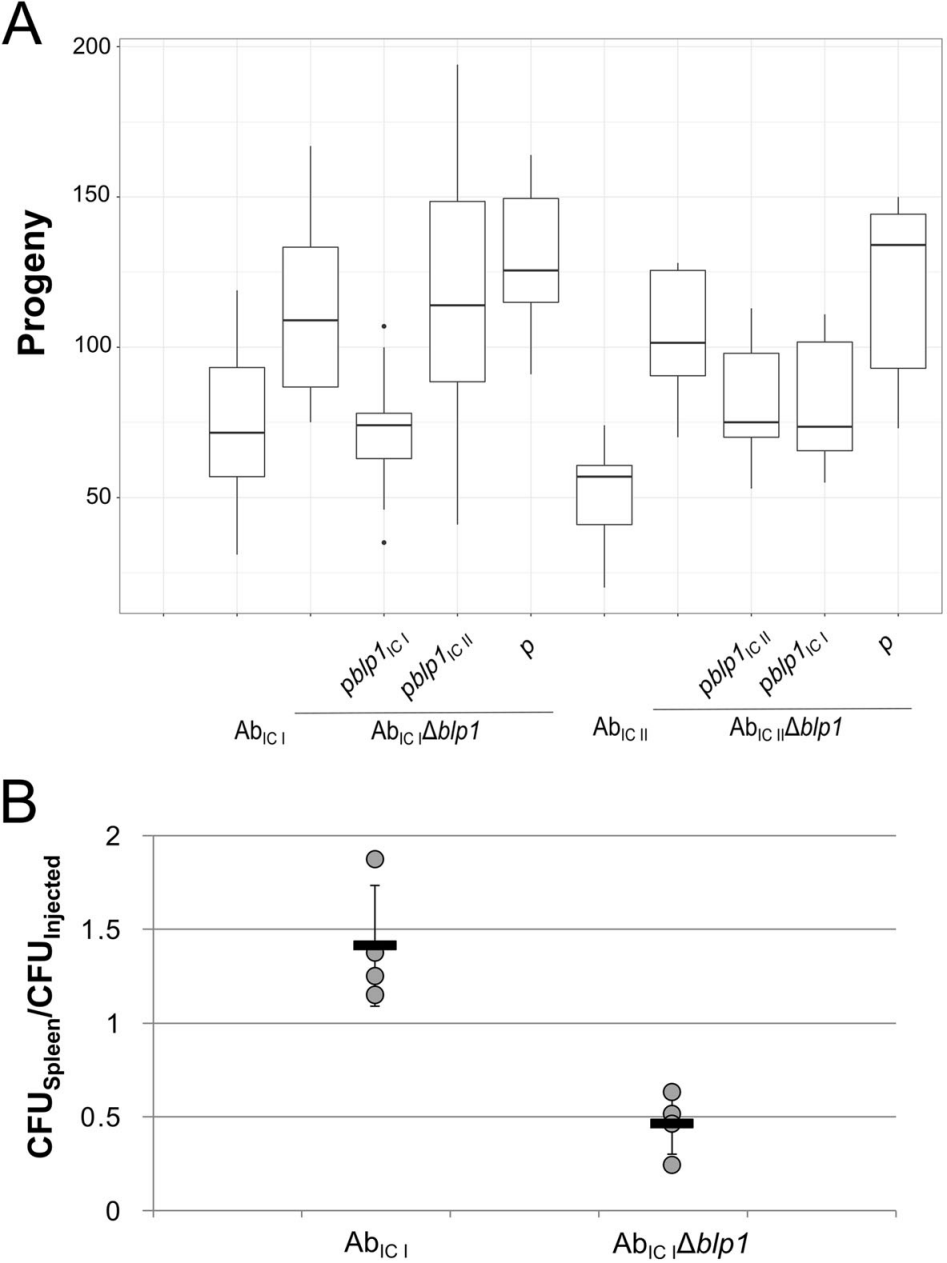
*A. baumannii* virulence in animal infection models. **a** – *C. elegans* fertility was evaluated after 3 days of nematodes growth in the presence of *A. baumannii* bacteria on NGM plates; box plot represents the count of nematodes progeny after incubation; data are given from at least two independent experiments, three plates were used in the each experiment; black lines represent medians and whiskers – minimum to maximum values. **b** – mice sepsis infection model represented as relative amount of *A. baumannii* CFUs in the spleens from BALB/c mice 6 h after infection compared to the injected amount of total CFUs (*n* = 4 mice per group); mice were intraperitoneally infected with 4 × 10^5^ CFU and 9.5 × 10^5^ CFU of Ab_IC I_ and Ab_IC I_Δ*blp1*, respectively; each dot represents one mouse, black lines indicate averages, whiskers represent standard deviations

The observed unequal role of *blp1* alleles in biofilm formation and cell adhesion prompted us to evaluate their importance in *A. baumannii* host colonization. For this purpose, *C. elegans* fertility model was applied as described in Materials and Methods section. As can be seen in Fig. 4a, *blp1* deletion in both Ab_IC I_ and Ab_IC II_ strains resulted in approximately 1.5-fold increase of worm progeny compared to that observed when *C. elegans* was infected with the parental strains, indicating reduced virulence in *blp1* deletion mutants.

Both *blp1* variants when present on the plasmids were able to restore impaired virulence of Ab_IC II_Δ*blp1 *strain manifested by the decrease in worm progeny numbers. Interestingly, the virulent properties of Ab_IC I_Δ*blp1* strain demonstrated towards *C. elegans*, were complemented with the cognate *blp1* allele only (Fig. 4a).

Next, we investigated the role of *blp1* gene in *A. baumannii* ability to colonize a vertebrate host. For this purpose, the Ab_IC I_ strain and its Δ*blp1* mutant were used for the infection. BALB/c mice were infected intraperitoneally and the bacterial burden in the spleens was examined upon 6 h after infection as described in Materials and Methods. As can be seen in Fig. 4b, approximately three-fold reduction in bacterial loads was observed in mice infected with Ab_IC I_Δ*blp1* strain compared with those treated with the parental strain indicating the importance of *A. baumannii blp1* gene in establishing infection within the host.

### Immunization with C-terminal fragment of Blp1 protein protects from the lethal *A*. *baumannii* infection in mice

Our observation that *A. baumannii* Blp1 proteins, encoded by *blp1* gene variants, share conservative C-terminal 160 amino acid fragment, led us to propose, that it could represent a suitable antigen candidate for investigation of immune-stimulatory properties of Blp1 protein. To increase the number of antigenic moieties displayed at the likely surface-exposed C-terminus of Blp1, 711 amino acids of C-terminal Blp1 from Ab_IC I_ (residues 2652–3362) with a N-terminus His-Tag was purified by affinity chromatography as described in Materials and Methods (Additional file 4: Figure S3).

**Fig. 5:**
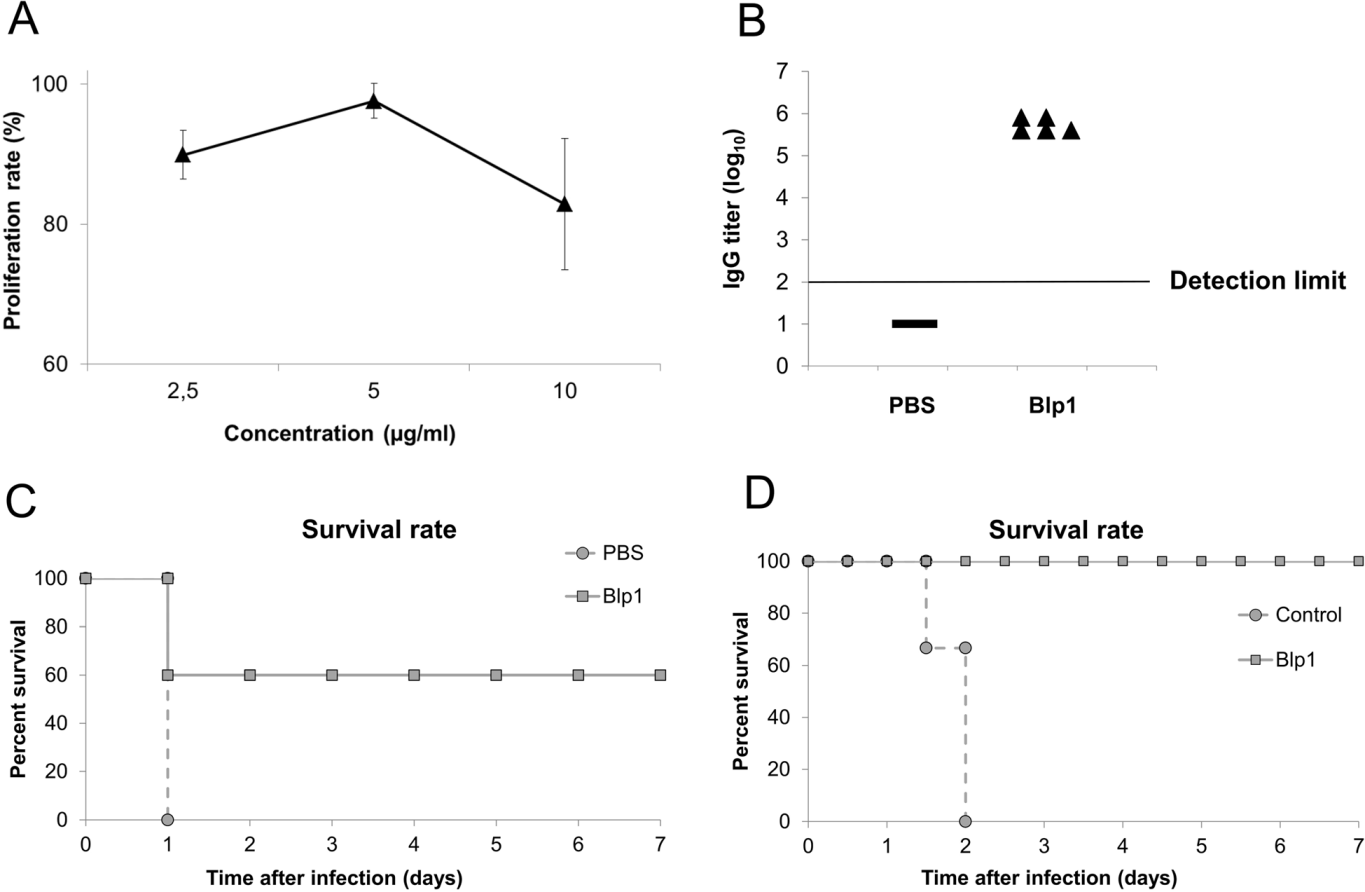
The effects of Blp1 C-terminal fragment as an immunization agent. **a** – proliferation rates of LL/2 cells were determined by MTS assay after 24 h of incubation with different concentrations of recombinant Blp1 fragment; error bars represent standard deviations from three repeats; **b** – titers of Blp1-specific IgG in mice were detected by ELISA (*n* = 5 mice per group); naïve sera obtained from mice, that received adjuvant with PBS were used as a control; horizontal line represents detection limit of the assay. **c** – mice survival rates after active vaccination using *A. baumannii* sepsis model; mice (*n* = 5 mice per group) were challenged intraperitoneally with 10^8^ CFUs of Ab_IC I_ and were monitored for 7 days; **d** – passive vaccination effect on the mice survival using *A. baumannii* sepsis model; control group received the serum obtained from mice, immunized with PBS and Freund’s adjuvant; mice (*n* = 3 mice per group) were challenged intraperitoneally with 5 × 10^7^ CFU of Ab_IC I_ and were monitored for 45 days

First, the cytotoxicity of purified recombinant Blp1 fragment has been evaluated using mouse lung epithelium LL/2 cells. The purified antigen demonstrated a mild suppressing effect on the growth of LL/2 cells in vitro in the concentration range from 2.5 to 10 μg/ml allowing the maintenance of approximately 90–83% viability of the cells for 24 h (Fig. 5a). Mild cytotoxicity of purified Blp1-specific antigen towards lung epithelium cells suggests that it represents a safe candidate antigen for vaccination.

For the active immunization, the 2 μg of the recombinant Blp1 fragment were injected into BALB/c mice (*n* = 5 per group) intramuscularly at the frequency of every 2 weeks, three times in total. At the fourth day after the last immunization, the blood samples were taken and obtained antisera were used for the determination of Blp1-specific-IgG titer by ELISA. The obtained results showed the induction of specific IgG response in the animal group immunized with Blp1 specific antigen compared to the control group (Fig. 5b). Then, on day 42nd, the intraperitoneal challenge with *A. baumannii* Ab_IC I_ strain (10^8^ CFU per mouse) has been undertaken and the mice survival rates were monitored for 7 days. The animal group, which received Blp1 specific antigen, demonstrated the 60% survival rate, whereas no survival in the control group was recorded (Fig. 5c).

For the passive immunization, the antisera, obtained from the mice immunized with Blp1 specific antigen and from the control group, treated with PBS, were used. Antisera were injected intraperitoneally into the naïve mice (*n* = 3 per group). Six hours later, animals were challenged with *A. baumannii* by inoculating 5 × 10^7^ CFUs of Ab_IC I_ strain. The survival was monitored for 45 days. As can be seen in the results presented in Fig. 5d, the group, which received antiserum against Blp1 specific antigen, yielded 100% survival rate, whereas group of mice, that received serum from the control group, resulted in 0% survival indicating an efficient protective immunity to *A. baumannii* infection, exerted by Blp1 specific antiserum.

### Antiserum against Blp1 C-terminal fragment induces opsonophagocytic killing of *A*. *baumannii* 

**Fig. 6:**
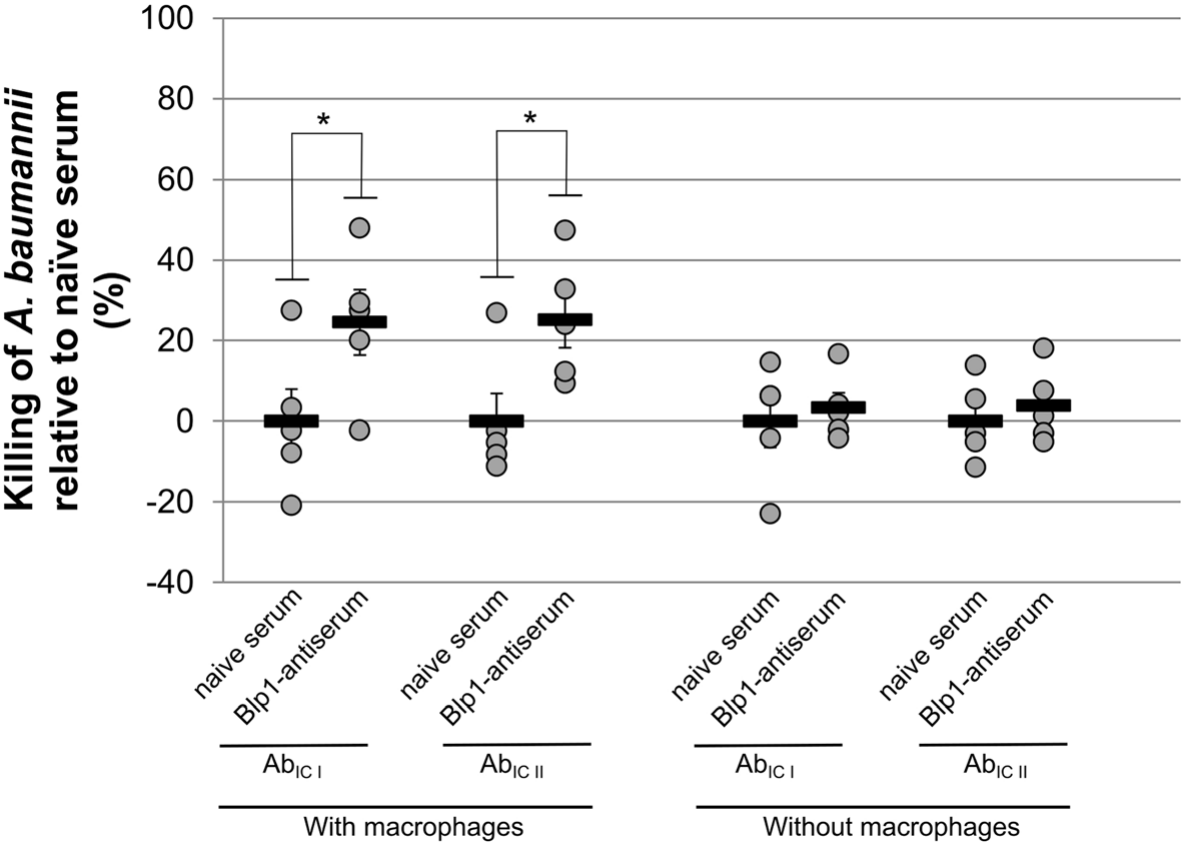
Blp1-antiserum-depended opsonophagocytic killing of *A. baumannii* IC I and IC II strains. The bactericidal killing activities of heat-treated Blp1-antiserum and heat-treated naïve serum were analyzed on *A. baumannii* Ab_IC I_ and Ab_IC II_ strains in the presence and absence of J774 macrophages; each dot represents one mouse, black lines indicate averages and whiskers represent standard errors; significance was assessed by *t*-test, (*P < 0.05)

To identify the main mechanism responsible for antimicrobial activity of obtained Blp1-antiserum, the *A. baumannii* Ab_IC I_ and Ab_IC II_ strains were incubated for 1 h with heat-treated (to inactivate complement components) Blp1-specific and naïve serum in the presence or absence of J744 macrophages (see Materials and Methods). The presence of macrophages increased killing against both Ab_IC I_ and Ab_IC II_ strains by approximately 20%, when inactivated Blp1-antiserum was present and compared to naïve serum (Fig. 6). In the absence of macrophages, killing efficiency of *A. baumannii* was negligible regardless the origin of inactivated antisera.

Therefore, an increase of opsonophagocytic killing of *A. baumannii* using heat-inactivated Blp1-antiserum indicates that the antimicrobial activity is macrophage-mediated and not complement-depended.

## Discussion

The *A. baumannii* Blp1 protein belongs to RTX (Repeats-in-toxin) domain-containing protein family [25], however its cellular location is not known. The presence of the T1SS recognition sequence at the very end of C-terminus indicates the secretion of Blp1 via the type I secretion system. The predicted structural organization of *A. baumannii* Blp1 protein such as the presence of multiple Big-like repeats, suggests that it displays properties, which are similar to those observed in extended bacterial fimbrial type adhesins [9, 12, 14]. Indeed, the requirement of *A. baumannii blp1* gene for the adhesion of IC I strain AYE to the A549 lung epithelium cells was recently reported [11]. We performed a homology search using Swiss-Model platform and the results revealed giant surface-exposed proteins such as SiiE from *Salmonella enterica* and *Mp*IBP from *Marinomonas primoryensis* as *A. baumannii* Blp1 structural homologues (data not shown). Based on the structural investigations, the features of potential adhesin, anchored on the surface of bacterium via its N-terminus have been proposed for *Salmonella enterica* SiiE protein [12]. Similarly, the N-terminal domains of *Mp*IBP protein have been suggested to anchor the ice and diatom binding adhesin to the outer membrane of M. *primoryensis* [14]. Our in silico analysis revealed a short (18 residues) α-helix as a potential transmembrane sequence at the N-terminus of *A. baumannii* Blp1 protein (residues 120–137), which could anchor the protein in *A. baumannii* outer membrane, however, additional investigations are needed to determine the exact mechanism of *A. baumannii* Blp1 immobilization on the surface of bacteria.

Our bioinformatic analysis did not reveal any specific adhesive modules at the C-terminus of *A. baumannii* Blp1, similar to those present at the C-terminus of its structural homolog such as in *M. primoryensis Mp*IBP [14]. However, this study demonstrated the surface adhesion-related properties of Blp1 protein that strongly indicate its role as a bacterial adhesin.

Consistent with the previous study using *A. baumannii* IC I strain AYE [11], the deletion of a large *blp1* gene affected the structure of biofilm formed by Ab_IC I_ strain of the common ST231 serotype. The Ab_IC I_Δ*blp1* mutant formed areas with reduced biofilm thickness and increased PI-stained layer. Similar phenotype of impaired biofilm formation was observed for *A. baumannii* IC I strain AB307–0294, which lacked gene coding for Bap, a giant surface-associated protein [26]. We have observed the PI-stained bacteria of Ab_IC I_Δ*blp1* strain at the initial phase of biofilm formation. The early accumulation of PI-stained bacteria might indicate an abnormal biofilm maturation, as a consequence of the absence of bacterial secreted factors needed for biofilm structure formation. The lack of *blp1* could result in liberation of eDNA, as an additional reinforcement material for the extracellular matrix [23]. Interestingly, the impairment of the biofilm formation was observed for Ab_IC I_Δ*blp1* mutant only, whereas the deletion of its counterpart in IC II strain had no effect suggesting a different role of *blp1* variants on the biofilm-related phenotype in these strains. Moreover, the same trait of different impact of *blp1* alleles was clearly seen in the adherence of IC I and IC II strains to lung epithelium cells. However, both *blp1* alleles were able to restore phenotypes at a similar level in adhesion-defective Ab_IC I_Δ*blp1* mutant. As the *blp1* gene transcription was confirmed in both IC I and IC II strains (Additional file 2: Figure S1), the observed differences could be explained by the redundancy of *blp1* for tested adhesion-related phenotype of IC II strain, possibly through the contribution of other extracellular components. The significant differences in biofilm formation were observed comparing *A. baumannii* IC I strain AB5075 and IC II strain ACICU [27]. The cohesion of type IV pili in *A. baumannii* ACICU strain was suggested as a reason of bacterial aggregates, similar to those observed in the biofilm of Ab_IC II_ strain.

In contrast to biofilm formation and adhesive properties, our study showed the importance of both *blp1* variants for the host colonization of respective strains and, in addition, the requirement of the cognate allele for the manifestation of this phenotype in IC I strain. Several studies have demonstrated the allele-dependent variations in virulent properties of bacterial pathogens [19, 28, 29]. Therefore, the Blp1 may possess yet unknown function required for host colonization, different from that mediating the adhesive properties, which appear to be redundant in IC II strain. The genomes of *A. baumannii* IC II strains were found to be more variable, frequently coding for new genes or genes with a higher sequence divergence, which increases a pan-genome ratio, compared to the genomes of IC I strains [15–17]. Given the fact, that IC II strains outspread the IC I strains in the hospital environment [30, 31], it is likely that the flexibility of genome, allowing to accumulate new variants and combination of genes, could be the source of the advantage demonstrated by *A. baumannii* IC II strains, rather than the evolvement of particular protein variants with increased virulent properties.

One of the most promising strategies against MDR *A. baumannii* infections is a development of vaccines [6, 32]. The capsular and surface polysaccharides were researched as potent immunization agents [33, 34], however, their high diversity among *A. baumannii* clinical strains [35] limits development of an universal vaccine. The inactivated whole *A. baumannii* cells and outer membrane vesicles (OMVs) also demonstrated the induction of immune-response using murine models [36–38]. Compared to the complex antigen mixtures, the pure proteins as vaccine antigens are more desirable due to the safety concerns [39]. Indeed, several *A. baumannii* outer membrane proteins have been used for vaccine research, mostly the abundant outer membrane proteins [40–42]. However, the limitation is that the vast majority of clinical *A. baumannii* strains possess a thick outer layer of polysaccharides (capsule), which efficiently protects pathogens from the host immunity [7, 8] by shielding antigens, present on the cell surface of the pathogen. Therefore, the surface-exposed proteins, penetrating the capsule layer, might show superiority as candidates for vaccine development [43].

The high molecular mass of *A. baumannii* Blp1 and adhesion-related phenotype observed for *blp1* gene, suggest that Blp1 could penetrate the capsular polysaccharide layer. Blp1 C-terminus possesses a conservative fragment consisting of 160 amino acids, and represents a common antigen displayed by the most prevalent *A. baumannii* strains circulating in the clinical environment. Therefore, the features of Blp1, discussed above, constitute a favorable combination for its role as an immunogen. Differently from the other proposed *A. baumannii* antigens [40–42], this study characterizes a highly prevalent *A. baumannii* antigen, which, due to its high molecular mass, could possibly penetrate the bacterial capsule. This is a substantial advantage considering therapeutic applications, since *A. baumannii* uses capsular polysaccharides as its major virulence factor against host defence system [7, 8]. Importantly, for the evaluation of Blp1 immunostimulatory properties, we have used a common *A. baumannii* strains ST231 (IC I) and ST208 (IC II) and demonstrated, using both active and passive immunizations, that Blp1-specific antigen is a suitable vaccine candidate. The Blp1-antiserum induced an opsonophagocytic killing of IC I and IC II strains, indicating the stimulation of an immune response against clonally disseminated MDR *A. baumannii*. It is worth to mention, that since the immunisation trials were performed with limited number of mice, there is a need for further investigations using larger amount of samples in order to extend this study.

## Conclusions

In this study we have investigated the *A. baumannii* surface-exposed protein Blp1 with an emphasis on its role in virulence, expressed by clinical *A. baumannii* strains and its suitability as a drug target. We have observed differences between IC strains, which should be taken into consideration, when the new drugs are investigated. Ideally, for the development of vaccine candidates, the most prevalent *A. baumannii* strains, currently circulating in the clinical environment should be used, since this opportunistic pathogen is well known for its genome variability.

## Methods

### Bacterial strains and growth conditions

Strains used in this study are listed in the Additional file 1: Table S1. Ab_IC I_ and Ab_IC II_ strains are representative strains corresponding to IC I and IC II, respectively. Strains were isolated from Lithuanian University of Health Sciences Kauno Klinikos Hospital in 2010 and assigned to IC I and IC II by trilocus sequence-based typing (3LST) as described previously [20]. Clinical *A. baumannii* strains were cultured on Luria-Bertani (LB) agar plates at 37 °C. Liquid cultures were inoculated in LB media overnight.

### PCR

One hundred twenty-two clinical *A. baumannii* strains belonging to either IC I (*n* = 72) or IC II (*n* = 50) clonal lineage were screened for the presence of *blp1* alleles. Conventional PCR was undertaken using primers listed in Additional file 1: Table S1. Tm was calculated based on the primer sequences.

For *blp1* gene transcription analysis, clinical *A. baumannii* strains were grown in LB medium for 16 h. Total RNA was extracted using GeneJET RNA Purification Kit (Thermo Fisher Scientific) according to the manufacturer recommendations. cDNA was synthesized using RevertAid First Strand cDNA Synthesis Kit (Thermo Fisher Scientific). qPCR was performed using primer pairs listed in Additional file 1: Table S1.

### Generation of *A*. *baumannii* Δ*blp1* mutants and complemented strains

Markerless gene deletion was performed as previously described [44]. Briefly, upstream and downstream regions of *A. baumannii blp1* gene were amplified using primers listed in Additional file 1: Table S1. The amplicons were joined with gentamicin resistance cassette *aac3I* by overlap PCR. The resulting DNA was cloned into pUC19_sacB plasmid yielding pUC19_sacB_UDblp1Gm plasmid (Additional file 1: Table S1). *A. baumannii* strains were transformed with the resulting plasmid by electroporation and colonies were selected on LB agar with gentamicin. Colonies were inoculated in LB medium for 4 h. Serial dilutions were plated on LB agar with 10% sucrose. For the complementation, the DNA comprising the *blp1* gene with an upstream region including a putative promoter region was amplified using primers listed in Additional file 1: Table S1 and DNA from Ab_IC I_ and Ab_IC II_ strains as a template. The resulting DNA fragments were cloned into pUC19_gm_AcORI plasmid and selected in *E. coli* JM107 strain (Additional file 1: Table S1). The Ab_IC I_Δ*blp1*and Ab_ICII_Δ*blp1*strains were transformed with the resulting plasmids p*blp1*_IC I_ and p*blp1*_IC II_, respectively, by electroporation and colonies were selected on LB agar with 30 μg/ml gentamicin. Generated mutants and recombinant constructs were confirmed by sequencing.

### Confocal laser scanning microscopy (CLSM)

For evaluation of biofilms, 1000-fold dilutions of overnight *A. baumannii* cultures were used for seeding into LB media. Biofilms were grown for 2 and 24 h at 37 °C without agitation. After growth in micro-titer plates, biofilms were stained for 2 h by the Filmtracer TM LIVE/DEAD® Biofilm Viability Kit (Thermo Fisher Scientific). The plate was then placed on the motorized stage of an inverted confocal microscope (TCS SP8 AOBS, Leica Microsystems) at the INRA-MIMA2 imaging platform as described by [45].

### Cell culture assays

Mouse epithelial LL/2 (LLC1) and mouse macrophages J774 cell lines were grown in Dulbecco’s modified Eagle’s medium (DMEM) (Gibco) supplemented with 10% fetal bovine serum (FBS) (Gibco) at 37 °C with 5% CO_2_. Adhesion experiments were performed as described by [8]. Bacterial adherence to the LL/2 cells was expressed as a percentage of the CFU of adhered bacteria compared to the total number of CFUs of the initial inoculum.

For the opsonophagocytic killing assay, mouse macrophages J774 were stimulated with 40 ng/ml *E. coli* LPS (Sigma-Aldrich) for 3 days. Macrophages (1 × 10^5^ cells/well) and *A. baumannii* strains (1 × 10^4^ CFU/well) were added into the wells along with the heat-treated mice serum (56 °C for 40 min for the inactivation of complement components) at the final dilution of 1:200. After 1 h incubation, the samples were serially diluted and seeded. Serum killing rates were counted by comparing the number of the reduced CFUs with those observed using naïve serum.

A cytotoxicity assay was performed by incubating LL/2 cells with recombinant Blp1 C-terminal fragment in DMEM supplemented with 10% FBS at 37 °C for 24 h. Then, 20 μl of MTS (Promega) solution was added to the wells and cells were incubated for 4 h at 37 °C. After incubation, OD_490_ was determined. The proliferation rate was counted by comparing OD_490_ values for Blp1 C-terminus fragment-treated and non-treated cells.

### *C. elegans* fertility assay

*A. baumannii* strains were investigated using *C. elegans* fertility model as described previously [8]. Overnight cultures of different *A. baumannii* strains were seeded on NGM medium. One L2 stage worm was placed over each *A. baumannii* strain and incubated at 21.5 °C. On the third day after infection worm progeny was determined by counting *C. elegans* worms.

### Cloning and protein purification

The DNA of Blp1 C-terminal fragment spanning 2652–3362 amino acids of *blp1* coding region was amplified using primer pair BldBamF/BlXhR (Additional file 1: Table S1). The resulting amplicon was cloned into pET-28b expression plasmid by fusing 6xHis tag sequence to the N-terminus of the recombinant protein. The resulting plasmid was sequenced and transformed into the expression host strain *E. coli* ArcticExpress (DE3) (Additional file 1: Table S1). Culture was grown in LB broth containing 40 μg/ml of kanamycin to OD_600_ of 0.5. Protein expression was induced by adding 0.5 mM of IPTG and incubating at 14 °C for 16 h. Cells were suspended in lysis buffer (20 mM NaH_2_PO_4_ pH 7.4, 500 mM NaCl, 20 mM imidazole) supplemented with protease inhibitor PMSF, disrupted by sonication and centrifuged 12,000 x g at 4 °C, for 30 min to remove insoluble material. Proteins were purified from soluble fraction by affinity chromatography, using 1 ml HisTrapHPTM nickel-Sepharose column (GE Healthcare). Proteins were eluted by a linear gradient using buffer 20 mM NaH_2_PO_4_ pH 7.4, 500 mM NaCl, 500 mM imidazole. The eluted fractions were desalted using Sephadex G–25 (GE Healthcare) column, exchanging to PBS buffer.

### ELISA

Antigen-specific ELISA was performed to quantify the anti-Blp1 antibody response in sera from vaccinated mice as described before [46]. 96-well Maxisorp plates (Nalge Nunc) were coated with 0.2 μg Blp1 fragment/well by incubation at 4 °C in PBS overnight. The titer of specific IgG was established using horseradish peroxidase substrate (Thermo Fisher Scientific) and determining the absorbance at 450 nm.

### Murine models

Eight to 12 weeks old female BALB/c mice were purchased from Institute of Biochemistry, Life Science Center (Vilnius University, Vilnius). The animals were maintained and used in accordance with the recommendations of the directive 2010/63/EU of the European Parliament and of the Council of 22 September 2010 on the protection of animals used for scientific purpose. Study was performed under permission of Lithuanian State Food and Veterinary Service no. G2–72.

A sepsis model was established as described previously [37]. Briefly, *A. baumannii* cultures were prepared by mixing the bacterial suspension with 5% of porcine mucin (w/v; Sigma-Aldrich). BALB/c mice (*n* = 4 per group) were injected intraperitoneally with 0.5 ml of the sample. The CFUs of the corresponding the bacterial loads were determined by plating sequential dilutions on LB plates.

For the immunization experiments, groups of BALB/c mice (*n* = 5) were immunized intramuscularly with 2 μg of recombinant Blp1 protein fragment. Immunization mixture was prepared by mixing the antigen with an equal volume of complete Freund’s adjuvant on the day 0 and with incomplete Freund’s adjuvant on the days 14 and 28. Control group was inoculated with PBS combined with Freund’s adjuvant. On the day 32, blood samples were collected and tested against immunogen using ELISA as described above. Mice were challenged intraperitoneally on the day 42 with 1 × 10^8^ CFU of Ab_IC I_ bacteria.

For the passive immunization, the 200 μL of antiserum was injected intraperitoneally into naïve mice (*n* = 3 per group). Control group received the serum obtained from mice, immunized with PBS and Freund’s adjuvant. After 6 h, 5 × 10^7^ CFU of Ab_IC I_ bacteria were injected intraperitoneally.

After experiments mice were sacrificed using cervical dislocation.

### Bioinformatic and statistical analysis

Bioinformatic analysis was undertaken using genomes obtained from NCBI database. Sequences alignments were made using TexShade tool. Statistical comparisons were performed using equal variance *t*-test.

## Supplementary information

**Additional file 1:** **Table S1.** Oligonucleotides, plasmids and strains used in this work.

**Additional file 2:** **Figure S1.** *blp1* gene expression in *A. baumannii* IC I and IC II strains.

**Additional file 3: Figure S2.** CLSM analysis of biofilms formed by the *A. baumannii* strains after 2 and 24 h of incubation.

**Additional file 4: Figure S3.** Expression and purification of the recombinant His-Blp1_2652–3362_ C-terminal fragment.

## Data Availability

The datasets used and analyzed during the current study are available from the corresponding author on reasonable request.

## Abbreviations

cDNA: complementary DNA
CFU: Colony-forming unit
CLSM: Confocal laser scanning microscopy
DMEM: Dulbecco’s modified Eagle’s medium
DNA: Deoxyribose nucleic acid
eDNA: extracellular DNA
ELISA: Enzyme-Linked ImmunoSorbent Assay
FBS: Fetal bovine serum
IC: International clone
IPTG: Isopropyl β-D-1-thiogalactopyranoside
LB: Luria-Bertani
LPS: Lipopolisacharide
MDR: Multidrug resistance
MLST: Multilocus sequence type
NGM: Nematode growth medium
OMV: Outer membrane vesicles
PBS: Phosphate-Buffered Saline
PCR: Polymerase chain reaction
PI: Propidium iodide
qPCR: quantitative PCR
RNA: Ribonucleic acid
RTX: Repeats-in-toxin
SDS-PAGE: Sodium dodecylsulfate polyacrylamide gel electrophoresis
