# Supplementary material for: Blp1 protein shows virulence-associated features and elicits protective immunity to Acinetobacter baumannii infection
Source: BMC Microbiol. 2019 Nov 21;19:259. doi: 10.1186/s12866-019-1615-3 (PMC6873735; doi:10.1186/s12866-019-1615-3)
Supplement: Supplementary file 1 — Additional file 1: Table S1. Oligonucleotides, plasmids and strains used in this work. [file 12866_2019_1615_MOESM1_ESM.pdf]

Supplemental Table S1. Oligonucleotides, plasmids and strains used in this work.

**Primers**	**Sequence (5'to 3')**	**Purpose**	**Source**
BaF	GCACCAAATGCAGCAACTCA	*blp1*$_{IC\ I}$ gene allele detection	This work
BaR	TGCTCGTTACAGAAAGCGGT		
BaF	GCACCAAATGCAGCAACTCA	*blp1*$_{IC\ II}$ gene allele detection	This work
Blp2R	TACTCGTTACGGAAGGTTCT		
BaprB	GATCAGTATGGTCAATTAT	Expression of *blp1* gene	This work
BapRaiR	CAGCGTGATCAACTACAACTAC		
Fjgms	AGGACAGAAATGCCTCGAC	*aac(3)-I* amplification	[1]
Rjgms	ATCTCGGCTTGAACGAATT		
Bav1	CATTACAATGCTTAAGCTA	*blp1* upstream region amplification; additional sequence underlined	This work
Bav2	CATATTAAACAATAATTTGCTTCCTTCATCGGTAGAAAC		
Bav3	GCAAATTATTGTTTAATATG	*blp1* downstream region amplification; additional sequence underlined	This work
Bav4	GTCGAGGCATTTCTGTCCTGGTTTAGCAATAGAACGGAT		
Bpat	AAGAGACTTTTAATAGGCGAT	*blp1* gene deletion confirmation with Bav4 primer	This work
BlpKlF	ACTGGAATTCCTATAAGACACTTAACTTATGAG	*blp1* gene with upstream region amplification	This work
BlpKlR	AGTCGGTACCAATTAACTCCATCAGACTTAGTC		
Aac3I_seqR	CGAAGTCGAGGCATTTCTGT	Confirmation of transformed bacteria	This work
AcORI_seqR	AGGCTGTTGATAACTTTTGGAA		
BldBamF	TTCTAGGATCCGAATATTGCTCCAGTAATT	*blp1*$_{2652\text{-}3362}$ cloning into expression vector; restrictions sites underlined	This work
BlXhR	TTCTACTCGAGTTAAACAATAATTTGCTGG		

**Plasmids**	**Relevant characteristics**	**Source**
pUC19_sacB	pUC19 derivative with *sacB* gene from *Bacillus* sp.; for the generation of markerless gene deletion mutants	[1]
pUC19_gm_AcORI	pUC19 derivative with *aac(3)-I* gentamicin aminoglycoside acetyltransferase cassette and ori site from *Acinetobacter*; for the complementation experiments	This work
pUC_sacB_UDblp1Gm	pUC19_sacB derivative with upstream and downstream regions of *A. baumannii* *blp1* gene and *aac(3)-I* gentamicin aminoglycoside acetyltransferase cassette from	This work

	clinical *A. baumanni* strain; for the generation of markerless gene deletion mutants	
p*blp1*$_{\text{IC I}}$	pUC19_gm_AcORI derivative with *blp1* gene along with upstream region (plausible promoter) from Ab$_{\text{IC I}}$ strain; for the complementation experiments	This work
p*blp1*$_{\text{IC II}}$	pUC19_gm_AcORI derivative with *blp1* gene along with upstream region (plausible promoter) from Ab$_{\text{IC II}}$ strain; for the complementation experiments	This work
pET-28b	Protein expression vector	Novagen
pET-His- Blp1$_{2652\text{-}3362}$	Blp1$_{2652\text{-}3362}$ expression plasmid, His-tag fused N-terminally to protein	This work

**Strains**	**Relevant characteristics**	**Source**
Acinetobacter baumannii Ab$_{\text{IC I}}$	Representative IC I clone strain[a]; MDR strain, gentamicin sensitive	[2]
Acinetobacter baumannii Ab$_{\text{IC II}}$	Representative IC II clone strain[a]; MDR strain, gentamicin sensitive	[2]
Ab$_{\text{IC I}}$*Δblp1*	*blp1* gene-negative mutant of *A. baumannii* strain Ab$_{\text{IC I}}$; markerless	This work
Ab$_{\text{IC II}}$*Δblp1*	*blp1* gene-negative mutant of *A. baumannii* strain Ab$_{\text{IC II}}$; markerless	This work
Ab$_{\text{IC I}}$*Δblp1*::p	Ab$_{\text{IC I}}$*Δblp1* strain with pUC19_gm_AcORI plasmid	This work
Ab$_{\text{IC II}}$*Δblp1*::p	Ab$_{\text{IC II}}$*Δblp1* strain with pUC19_gm_AcORI plasmid	This work
Ab$_{\text{IC I}}$*Δblp1::*p*blp1*$_{\text{IC I}}$	Ab$_{\text{IC I}}$*Δblp1* strain complemented with p*blp1* $_{\text{IC I}}$	This work
Ab$_{\text{IC I}}$*Δblp1::*p*blp1* $_{\text{IC II}}$	Ab$_{\text{IC I}}$*Δblp1* strain complemented with p*blp1* $_{\text{IC II}}$	This work
Ab$_{\text{IC II}}$*Δblp1::*p*blp1* $_{\text{IC I}}$	Ab$_{\text{IC II}}$*Δblp1* strain complemented with p*blp1* $_{\text{IC I}}$	This work
Ab$_{\text{IC II}}$*Δblp1::*p*blp1* $_{\text{IC II}}$	Ab$_{\text{IC II}}$ *Δblp1* strain complemented with p*blp1* $_{\text{IC II}}$	This work
Escherichia coli OP50	Wild type, bacterial food source for *C. elegans*	[3]
E. coli JM107	endA1 glnV44 thi-1 relA1 gyrA96 Δ(lac-proAB) [F_ traD36 proAB+ lacIq lacZΔM15] hsdR17(RK- mK+) λ-	[4]
E. coli ArcticExpress (DE3)	B F$^-$ ompT hsdS(r_B^- m_B^-) dcm$^+$ Tet^r gal λ(DE3) endA Hte [cpn10 cpn60 Gent^r]	Thermo Fisher Scientific

[a] Strains were assigned to IC I and IC II by trilocus sequence-based typing (3LST) as described previously [2]; Strains were isolated from Lithuanian University of Health Sciences Kauno Klinikos Hospital in 2010.

[1] Skerniškytė J, Krasauskas R, Péchoux C, Kulakauskas S, Armalytė J, Sužiedėlienė E. Surface-related features and virulence among *Acinetobacter baumannii* clinical isolates belonging to international clones I and II. Front. Microbiol. 2019;9:3116.

[2] Povilonis J, Seputiene V, Krasauskas R, Juskaite R, Miskinyte M, Suziedelis K, et al. Spread of carbapenem-resistant *Acinetobacter baumannii* carrying a plasmid with two genes encoding OXA-72 carbapenemase in Lithuanian hospitals. J Antimicrob Chemother. 2013;68(5):1000-6.

[3] Brenner S. The genetics of *Caenorhabditis elegans*. Genetic. 1974; 77: 71–94;

[4] Yanisch-Perron C, Vieira J, Messing J. Improved M13 phage cloning vectors and host strains: nucleotide sequences of the M13mp18 and pUC19 vectors. Gene. 1985;33(1):103-19. Erratum in: Gene. 1992;114(1):81-3.
