# Supplementary material for: Blp1 protein shows virulence-associated features and elicits protective immunity to Acinetobacter baumannii infection
Source: BMC Microbiol. 2019 Nov 21;19:259. doi: 10.1186/s12866-019-1615-3 (PMC6873735; doi:10.1186/s12866-019-1615-3)
Supplement: Supplementary file 2 — Additional file 2: Figure S1. blp1 gene expression in A. baumannii IC I and IC II strains. [file 12866_2019_1615_MOESM2_ESM.pdf]

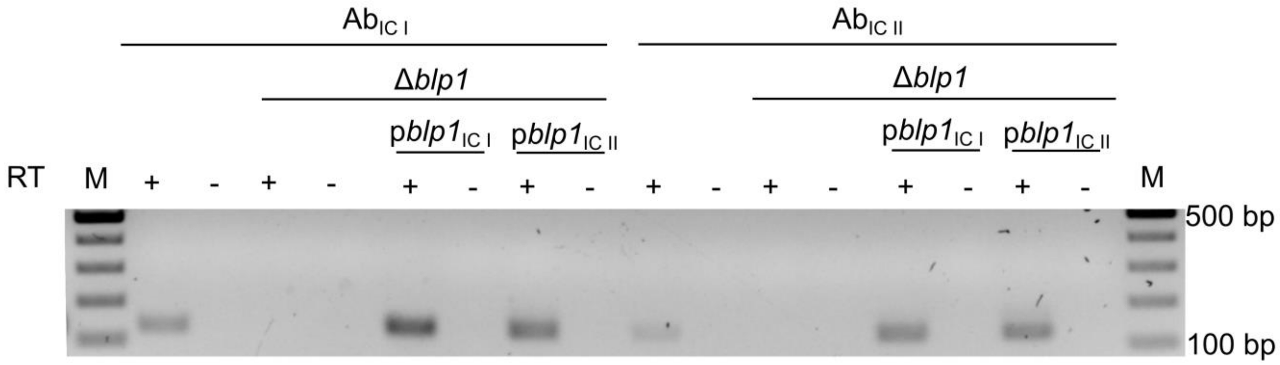

Supplemental Fig. S1. *blp1* gene expression in *A. baumannii* IC I and IC II strains. Amplicons of *blp1* fragment generated using cDNA obtained from *A. baumannii* strains Ab$_{IC\ I}$ and Ab$_{IC\ II}$, were fractionated on 1% agarose gel; RT +/- indicates the addition of reverse transcriptase during the synthesis of cDNA; M – GeneRuler Mix (SM0331, ThermoFisher Scientific)
