# Supplementary material for: Blp1 protein shows virulence-associated features and elicits protective immunity to Acinetobacter baumannii infection
Source: BMC Microbiol. 2019 Nov 21;19:259. doi: 10.1186/s12866-019-1615-3 (PMC6873735; doi:10.1186/s12866-019-1615-3)
Supplement: Supplementary file 3 — Additional file 3: Figure S2. CLSM analysis of biofilms formed by the A. baumannii strains after 2 and 24 h of incubation. [file 12866_2019_1615_MOESM3_ESM.pdf]

After 2 hours of incubation:

$Ab_{IC\ I}$

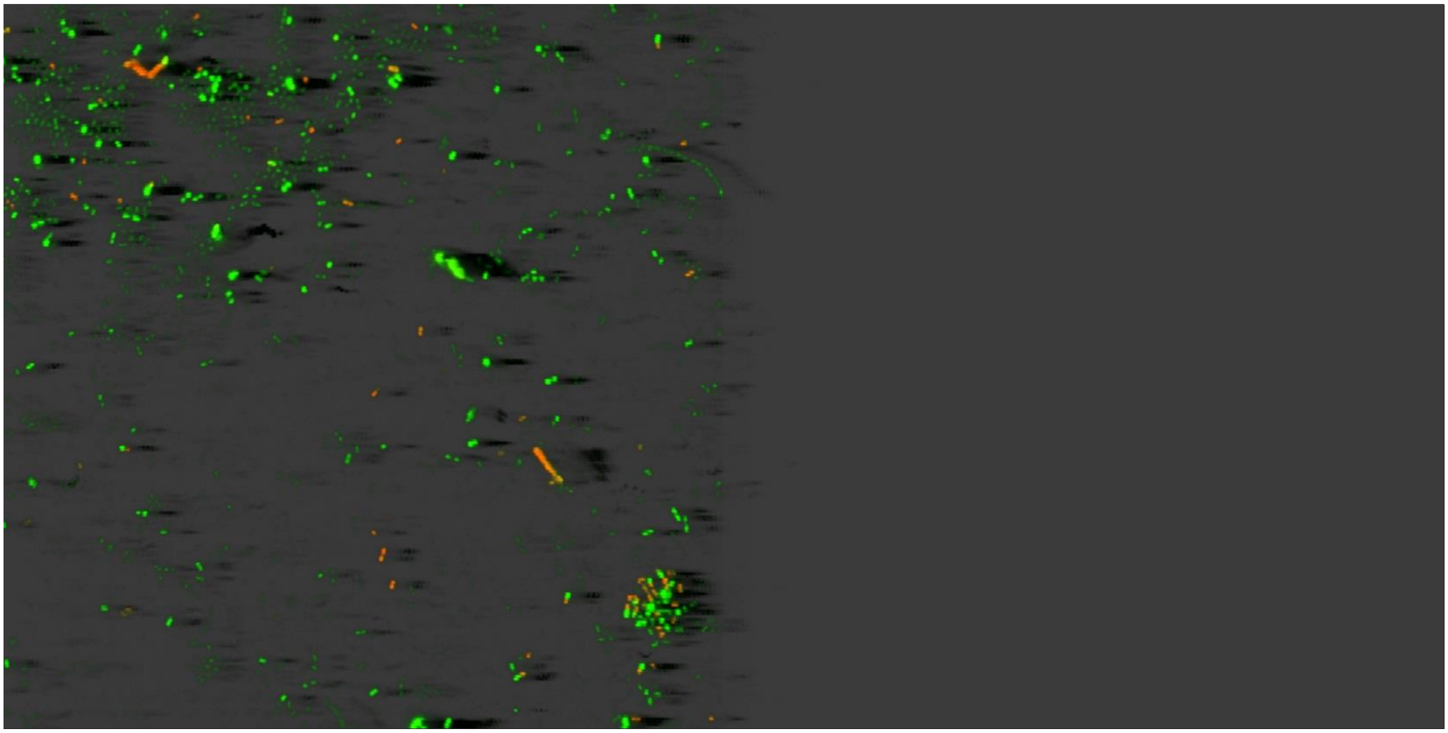

$Ab_{IC\ I}\Delta$*blp1*

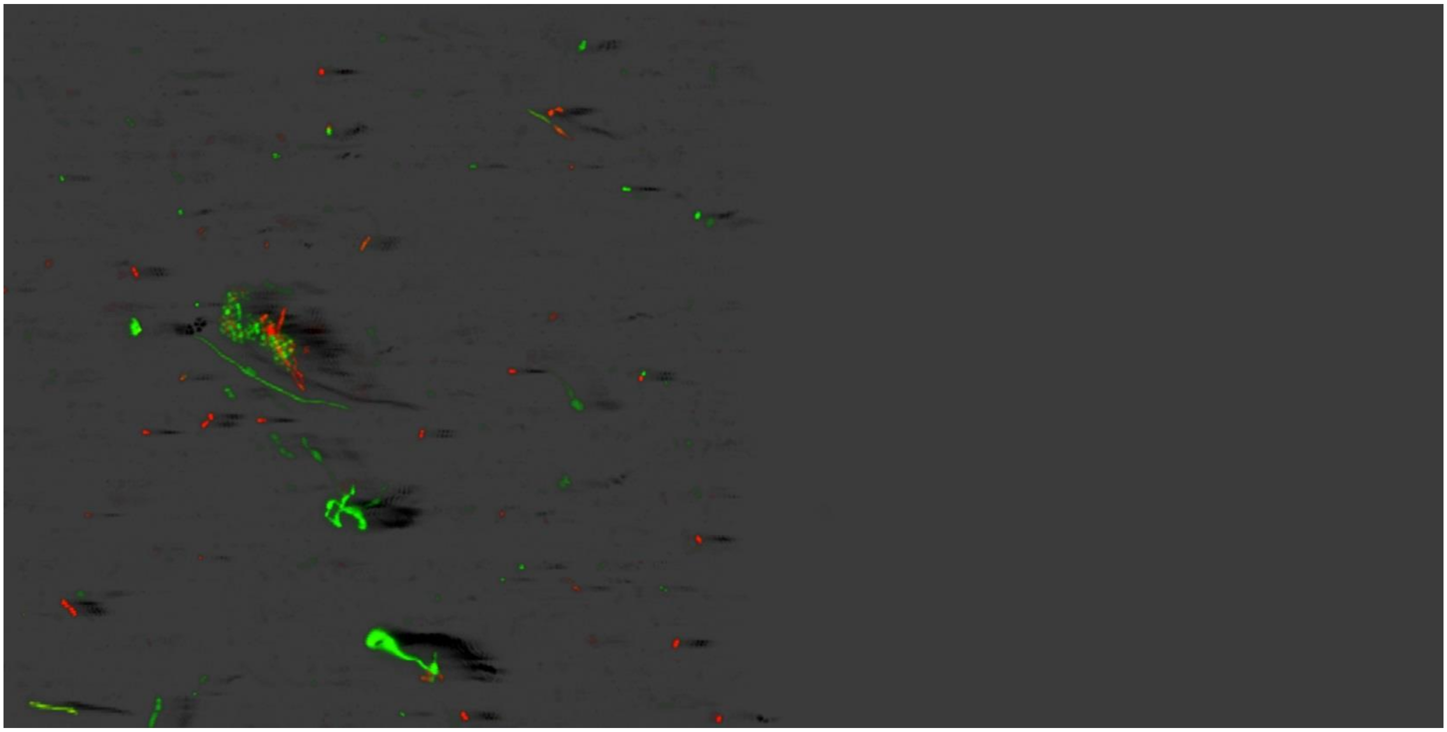

$Ab_{IC\ I}\Delta$*blp1*::p*blp1*$_{IC\ I}$

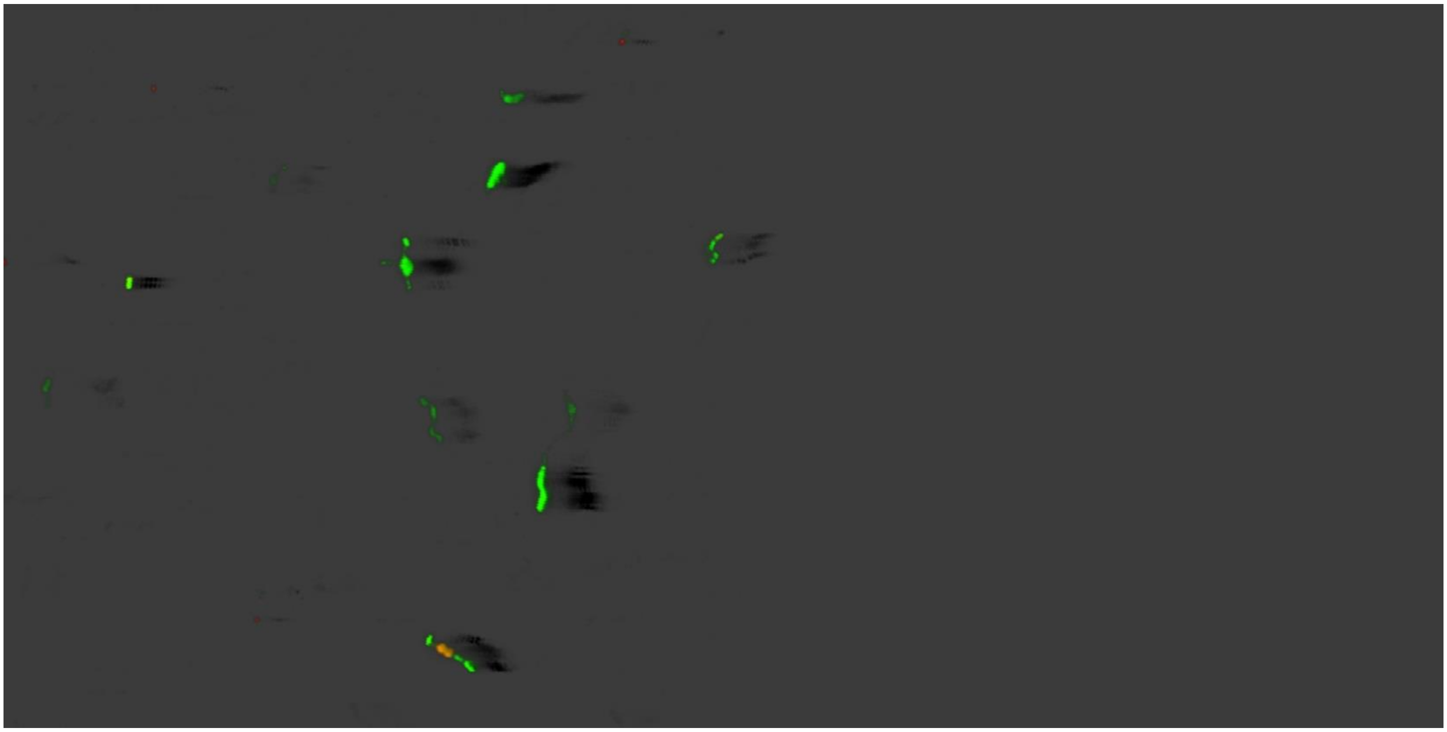

$Ab_{IC\ I}\Delta$*blp1*::p*blp1*$_{IC\ II}$

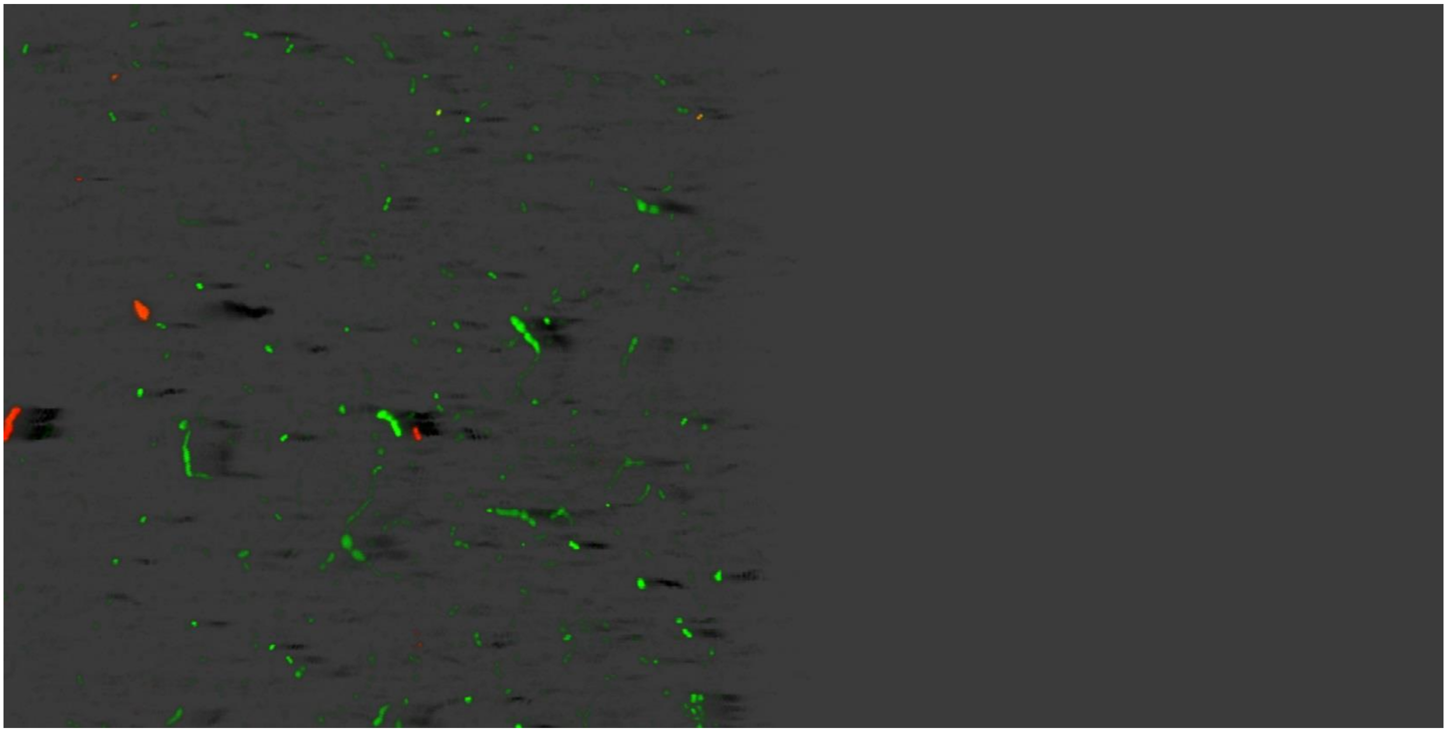

$Ab_{IC\ I}$Δ*blp1*::p

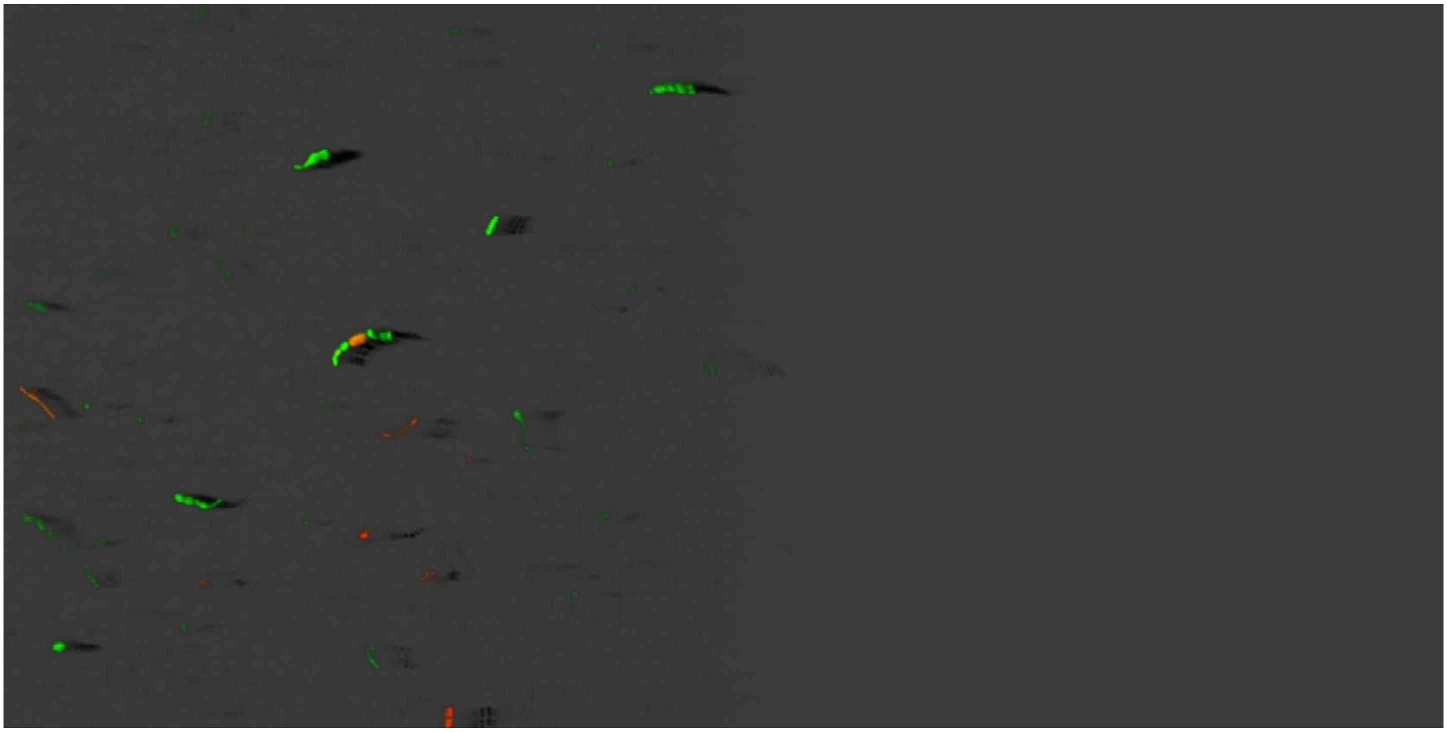

$Ab_{IC\ II}$

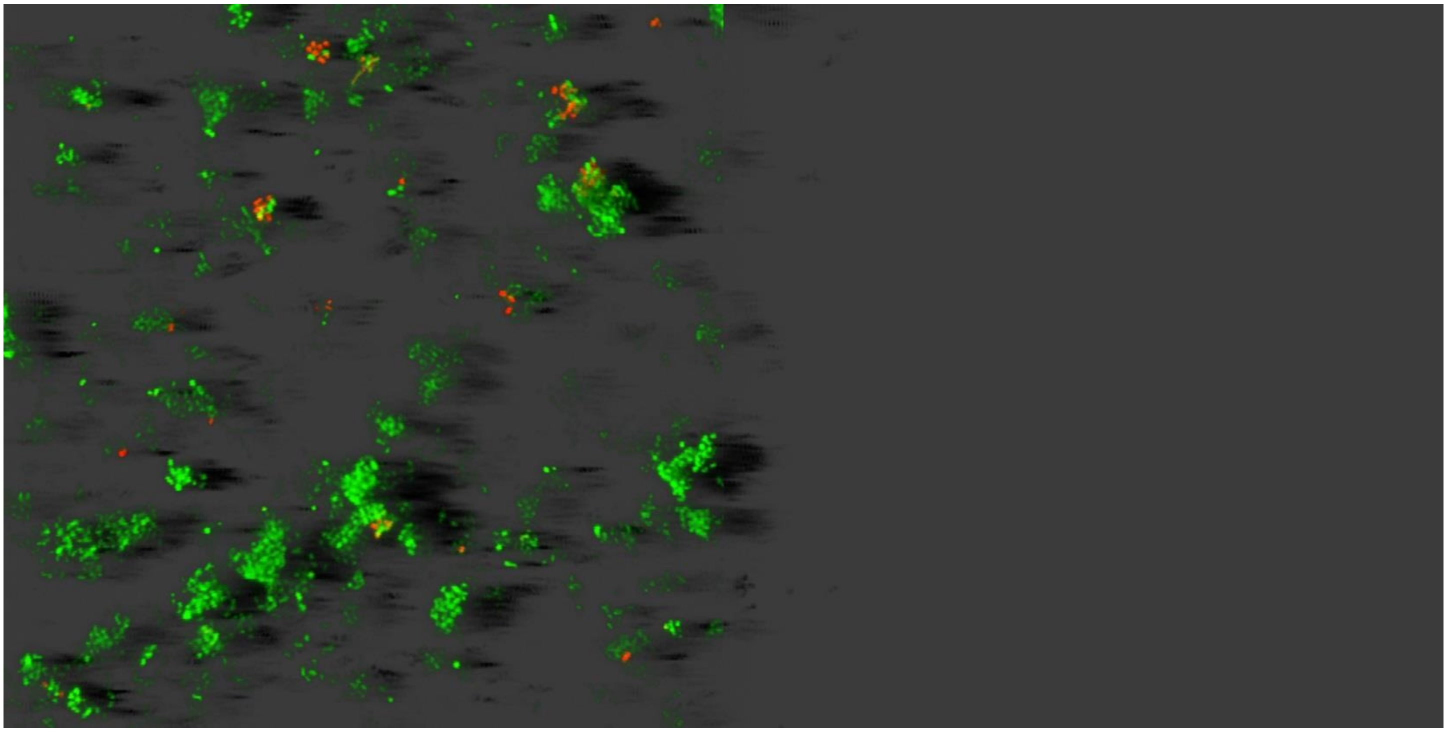

$Ab_{IC\ II}\Delta$*blp1*

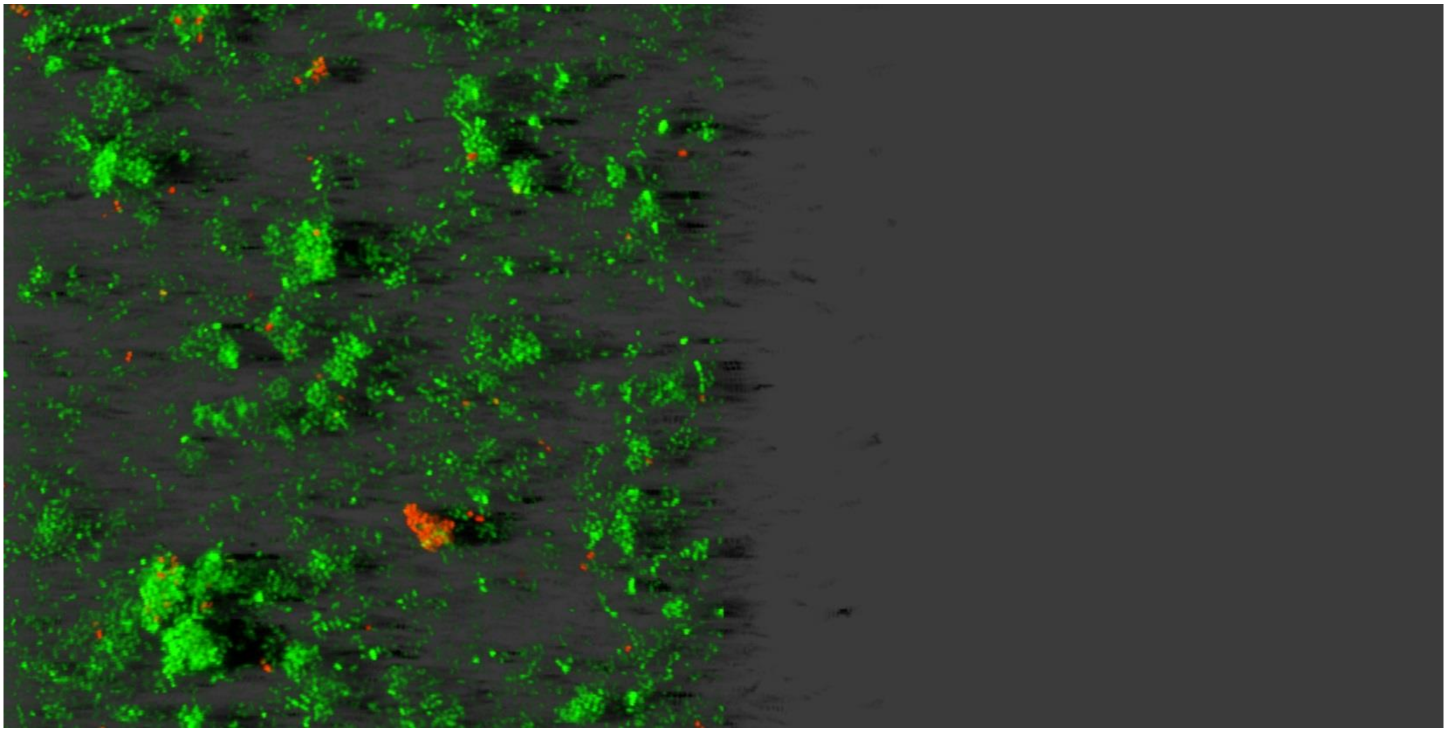

$Ab_{IC\ II}\Delta$*blp1*::p*blp1*$_{IC\ II}$

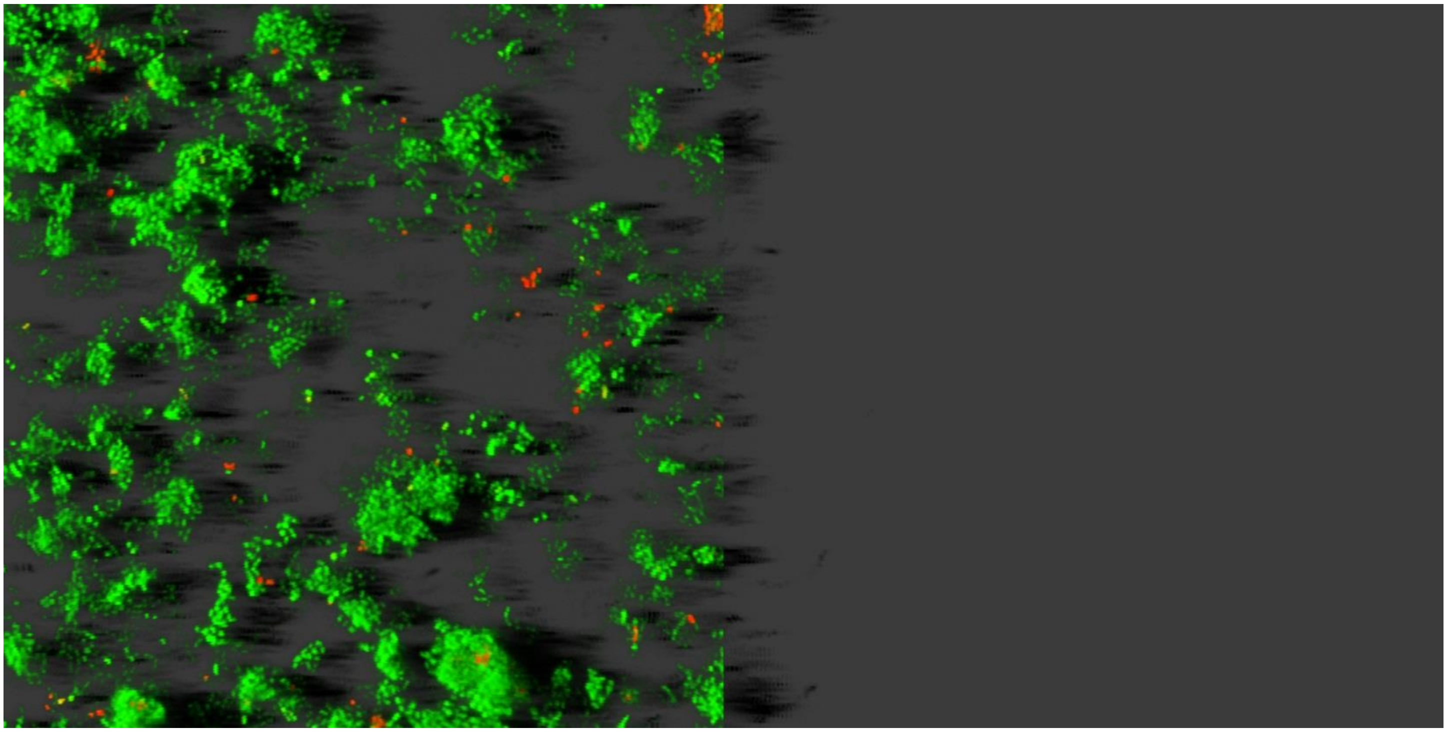

Ab$_{IC\ II}$Δ*blp1*::p*blp1*$_{IC\ I}$

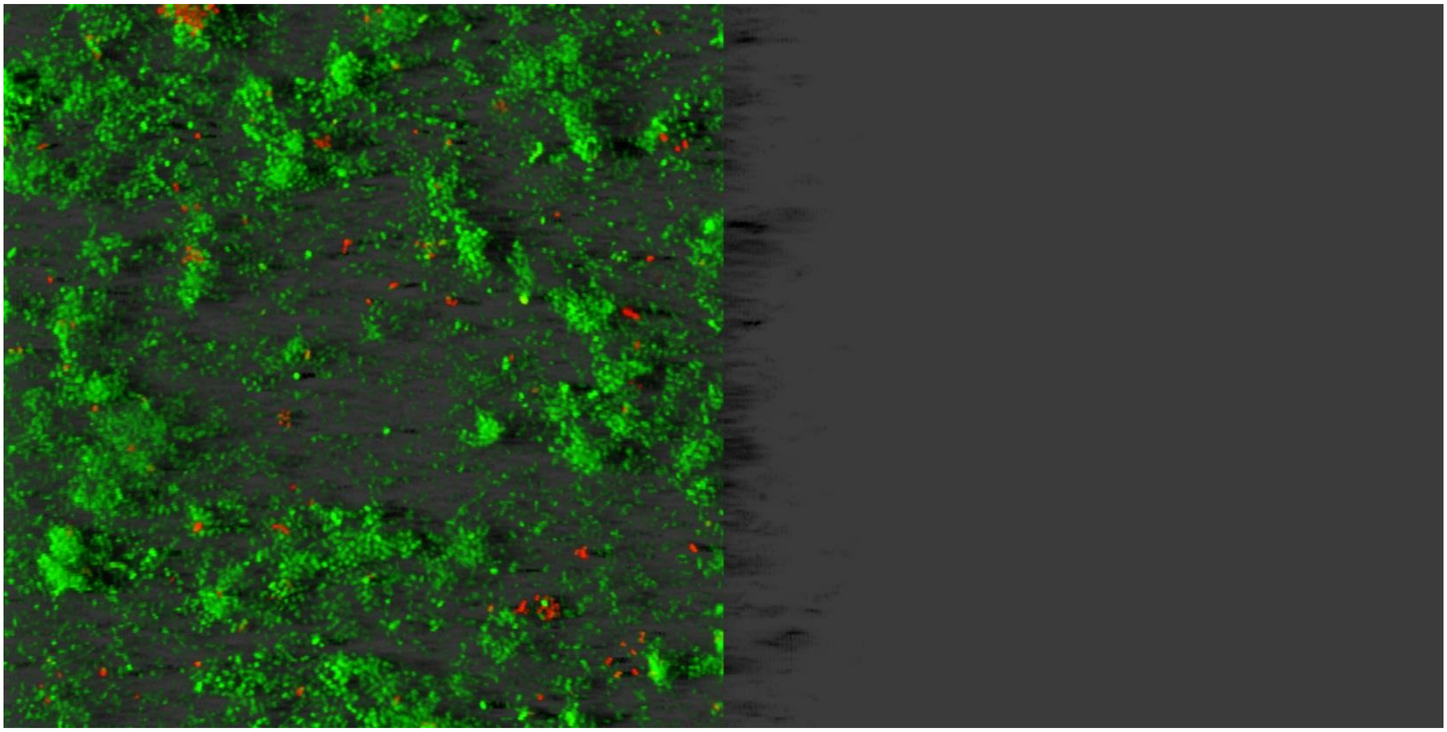

Ab$_{IC\ II}$Δ*blp1*::p

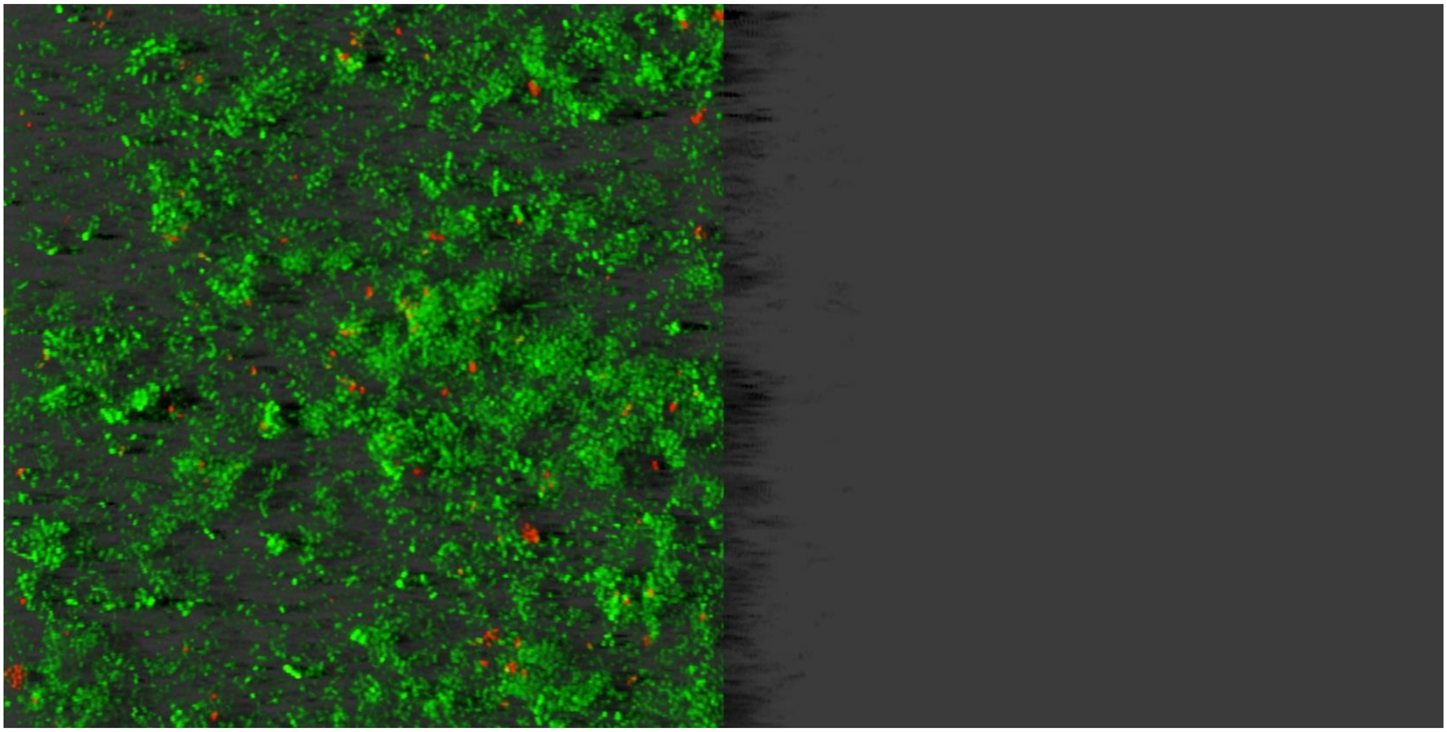

After 24 hours of incubation:

$Ab_{IC\ I}$

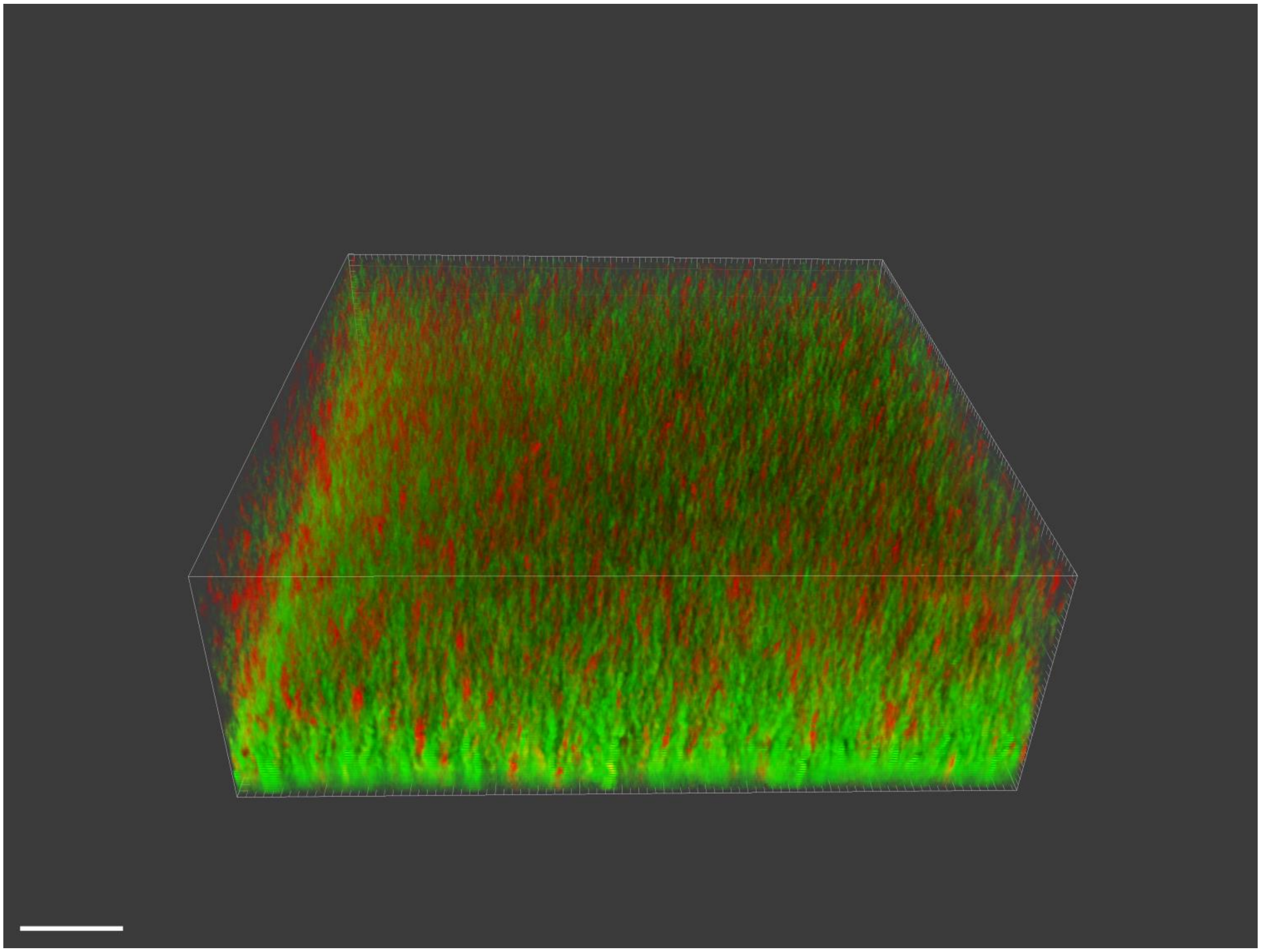

Ab$_{\text{IC I}}$Δ*blp1*

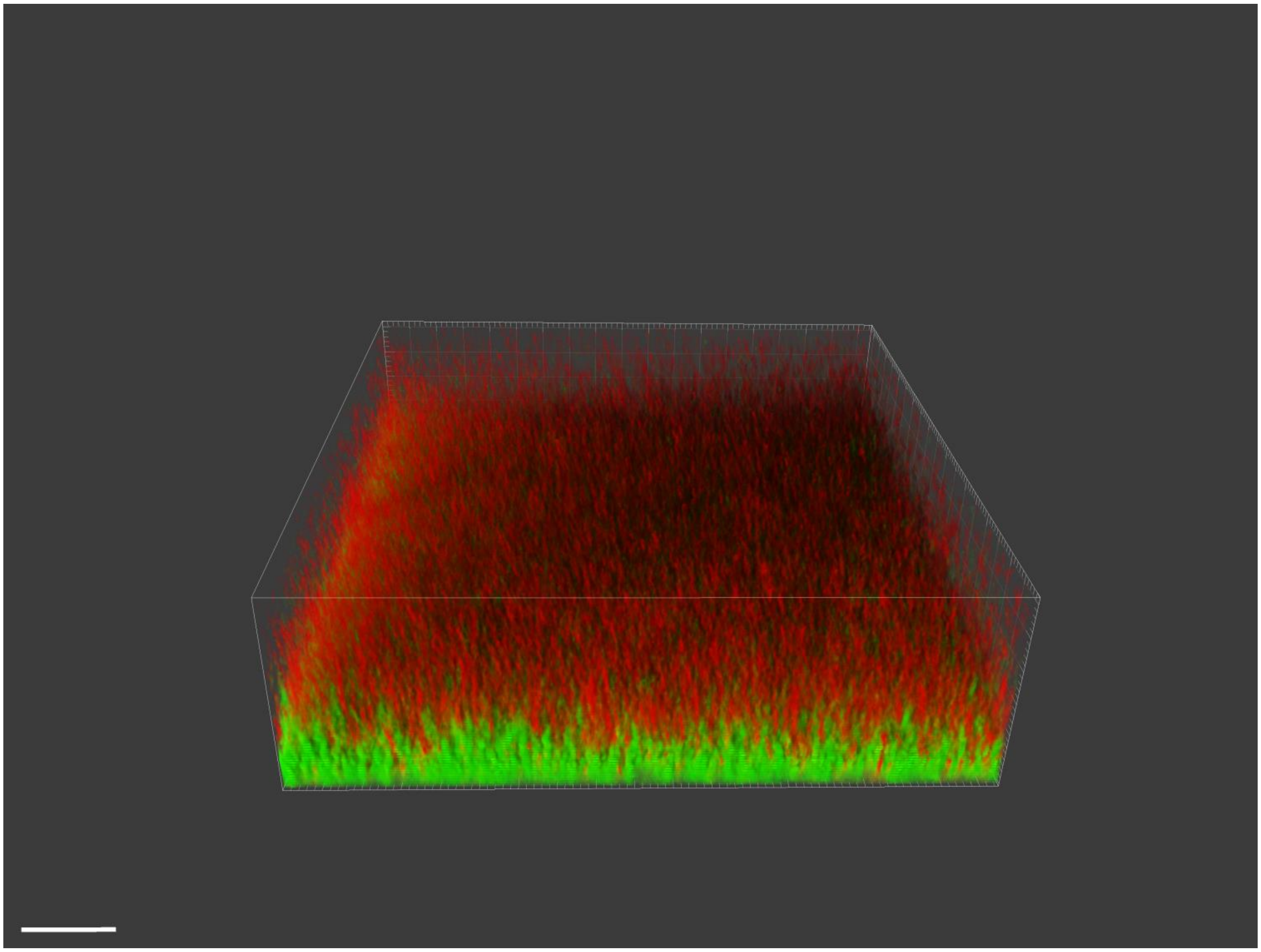

$Ab_{IC\ I}\Delta$*blp1*::p*blp1*$_{IC\ I}$

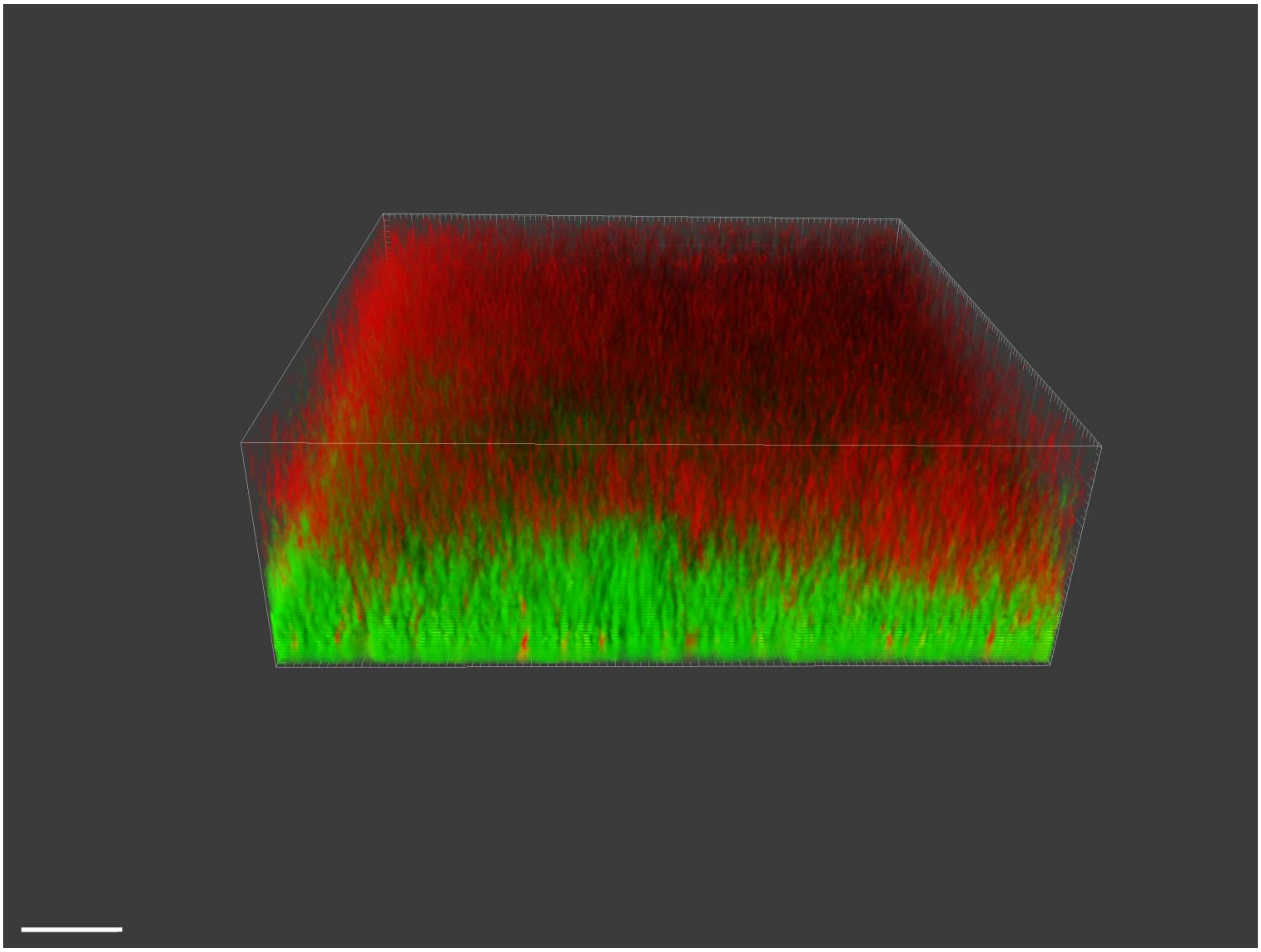

$Ab_{IC\ I}\Delta$*blp1*::p*blp1*$_{IC\ II}$

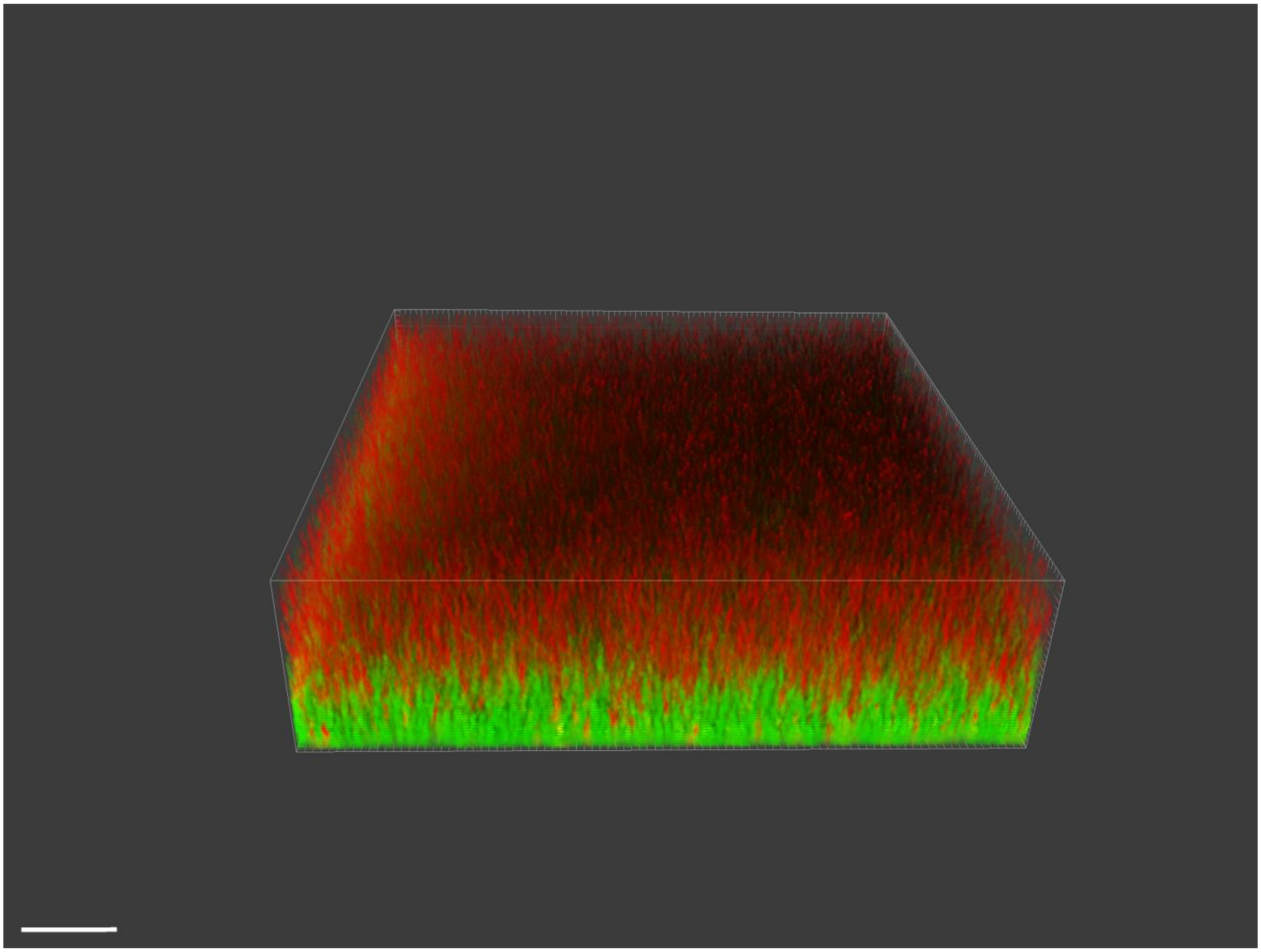

$Ab_{IC\ I}\Delta$*blp1*::p

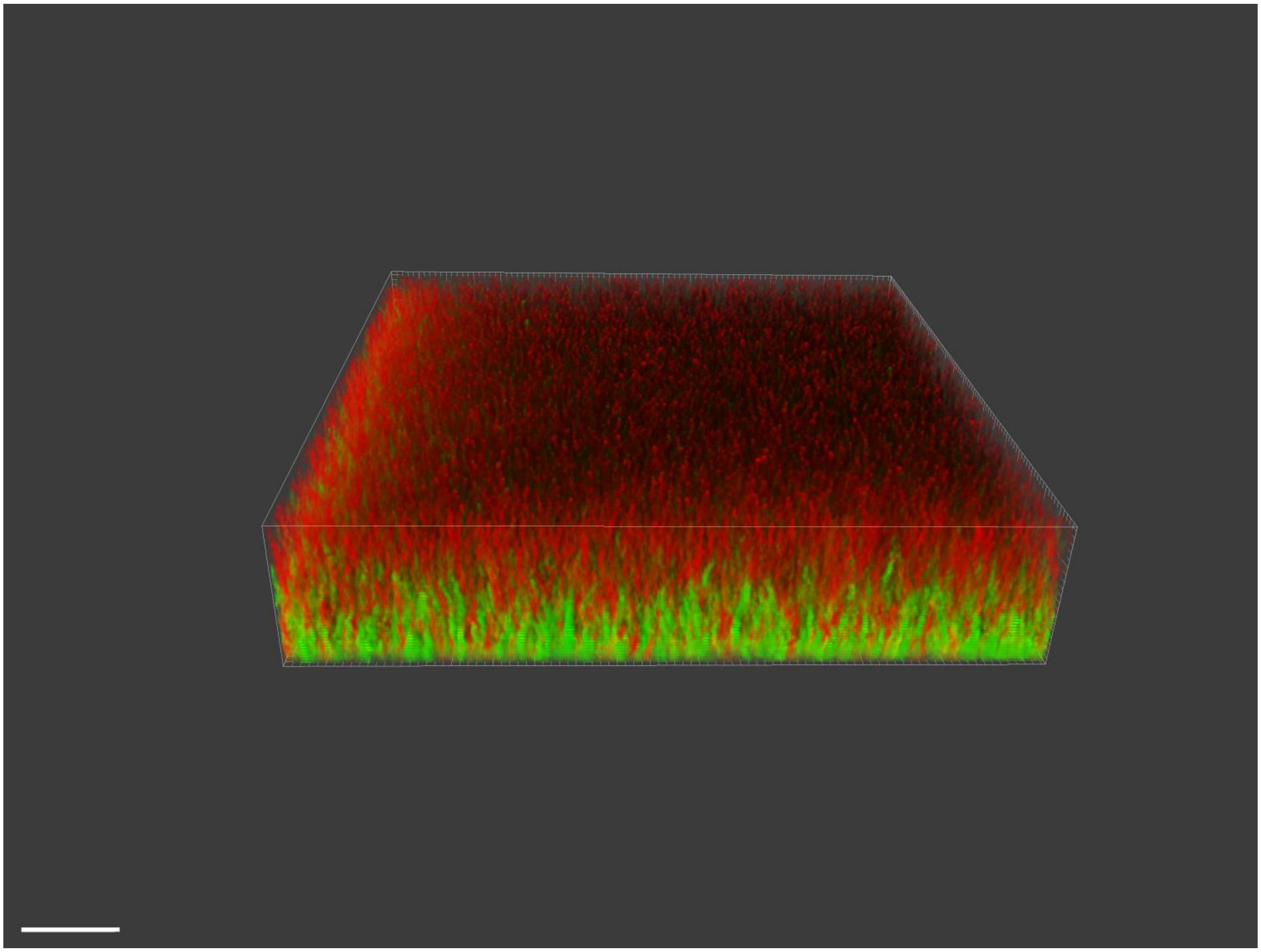

$Ab_{IC\ II}$

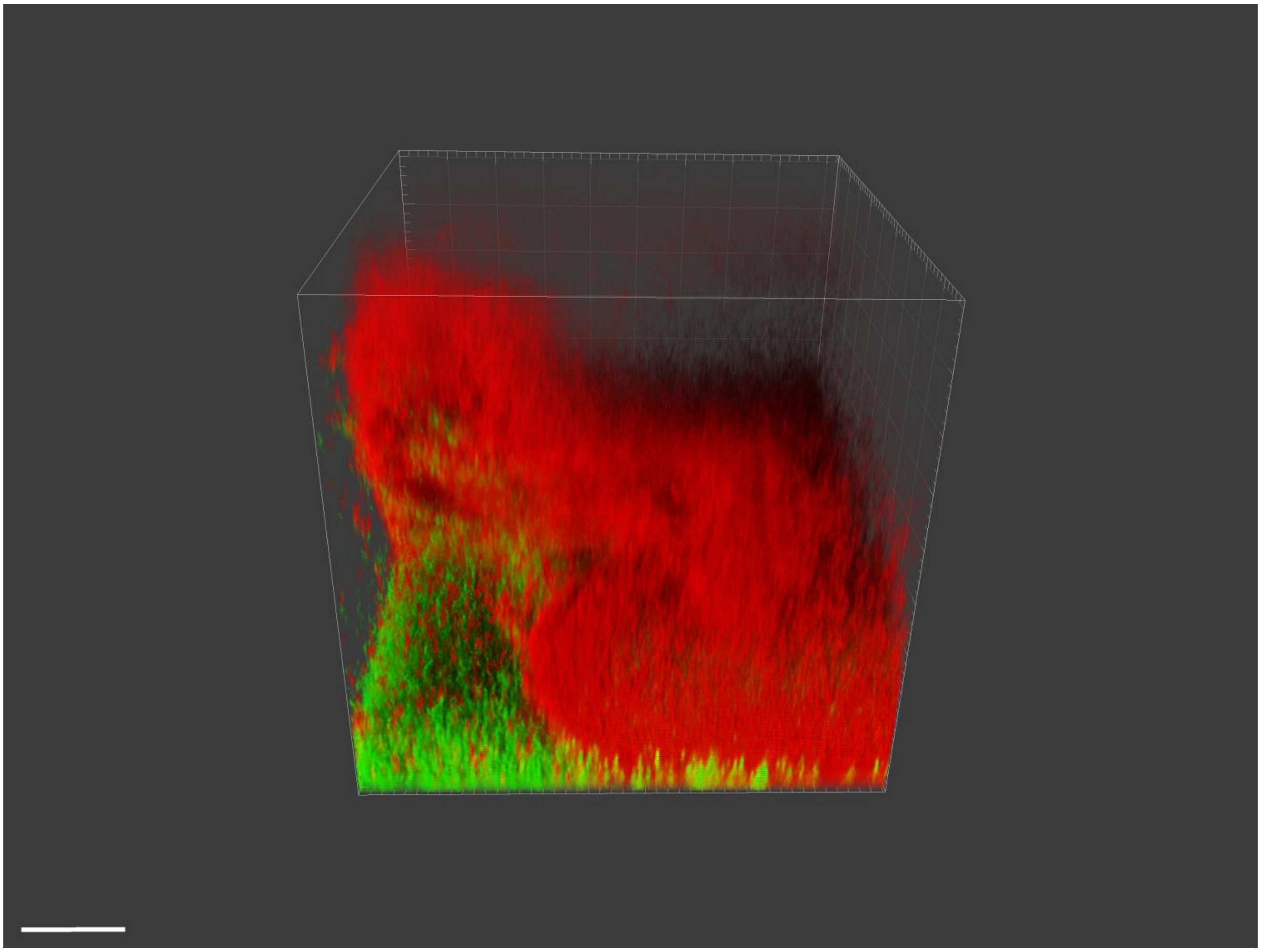

$Ab_{IC\ II}\Delta$*blp1*

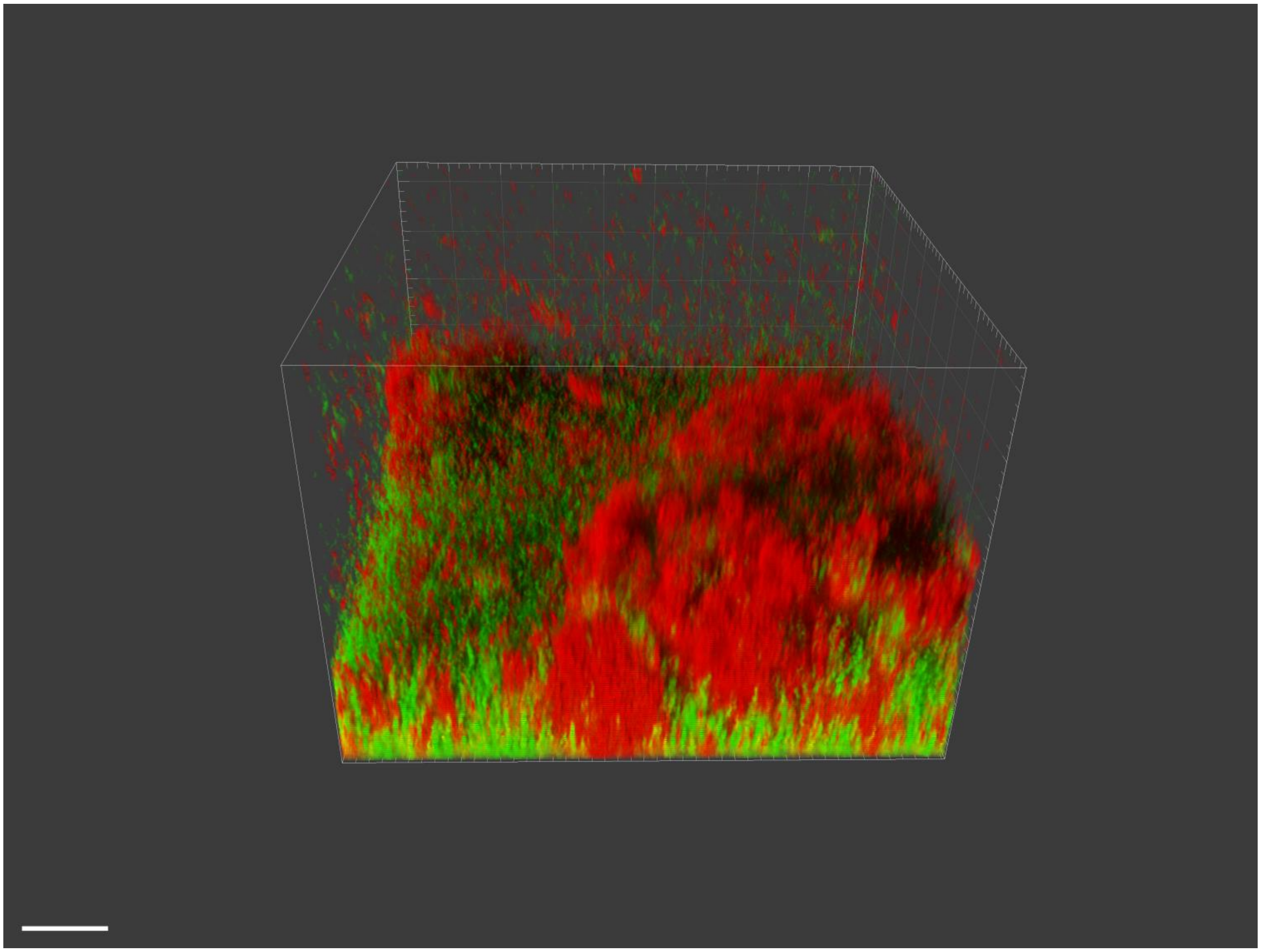

Ab$_{\text{IC II}}$Δ*blp1*::p*blp1*$_{\text{IC II}}$

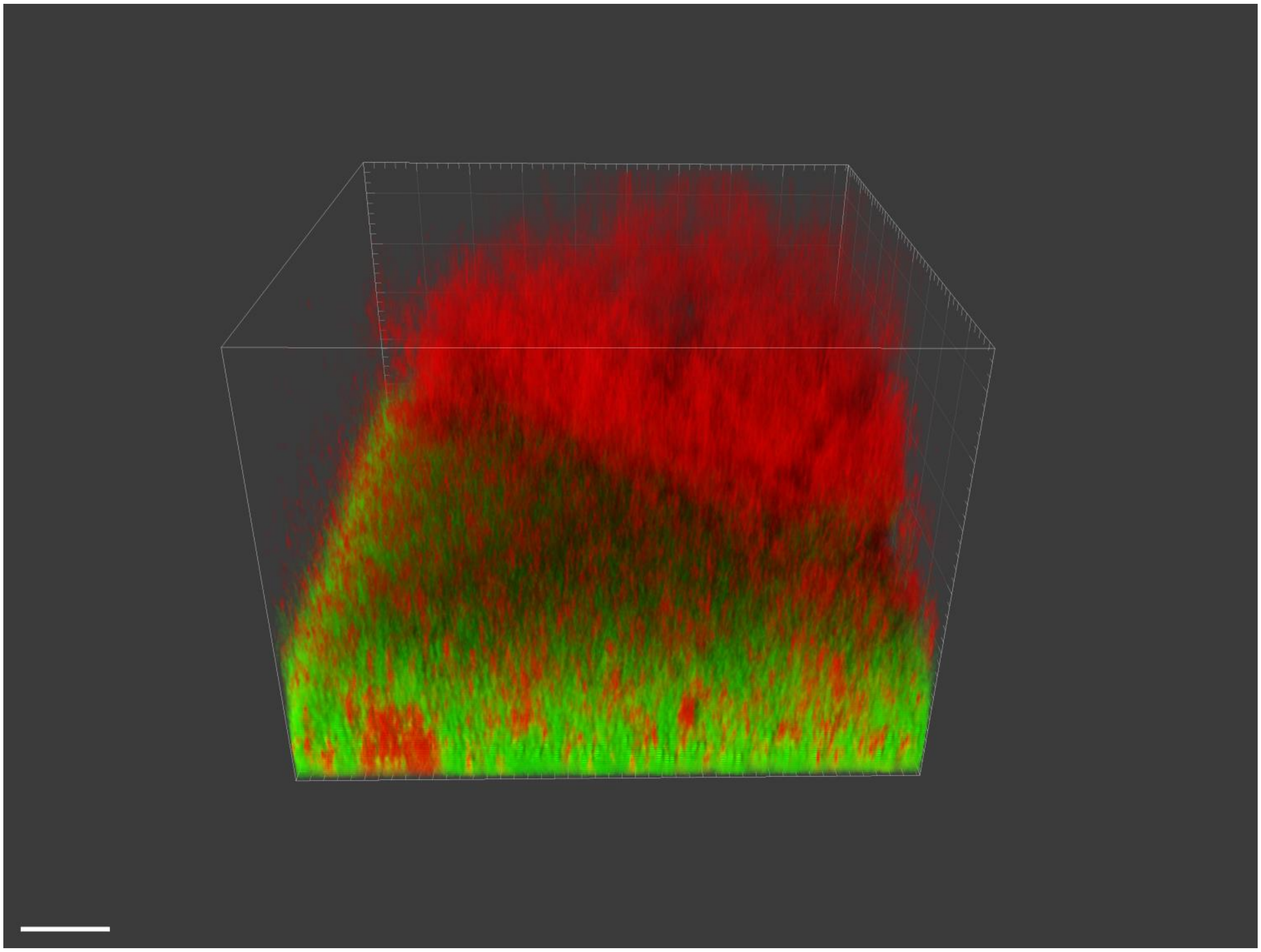

$Ab_{IC\ II}\Delta$*blp1*::p*blp1*$_{IC\ I}$

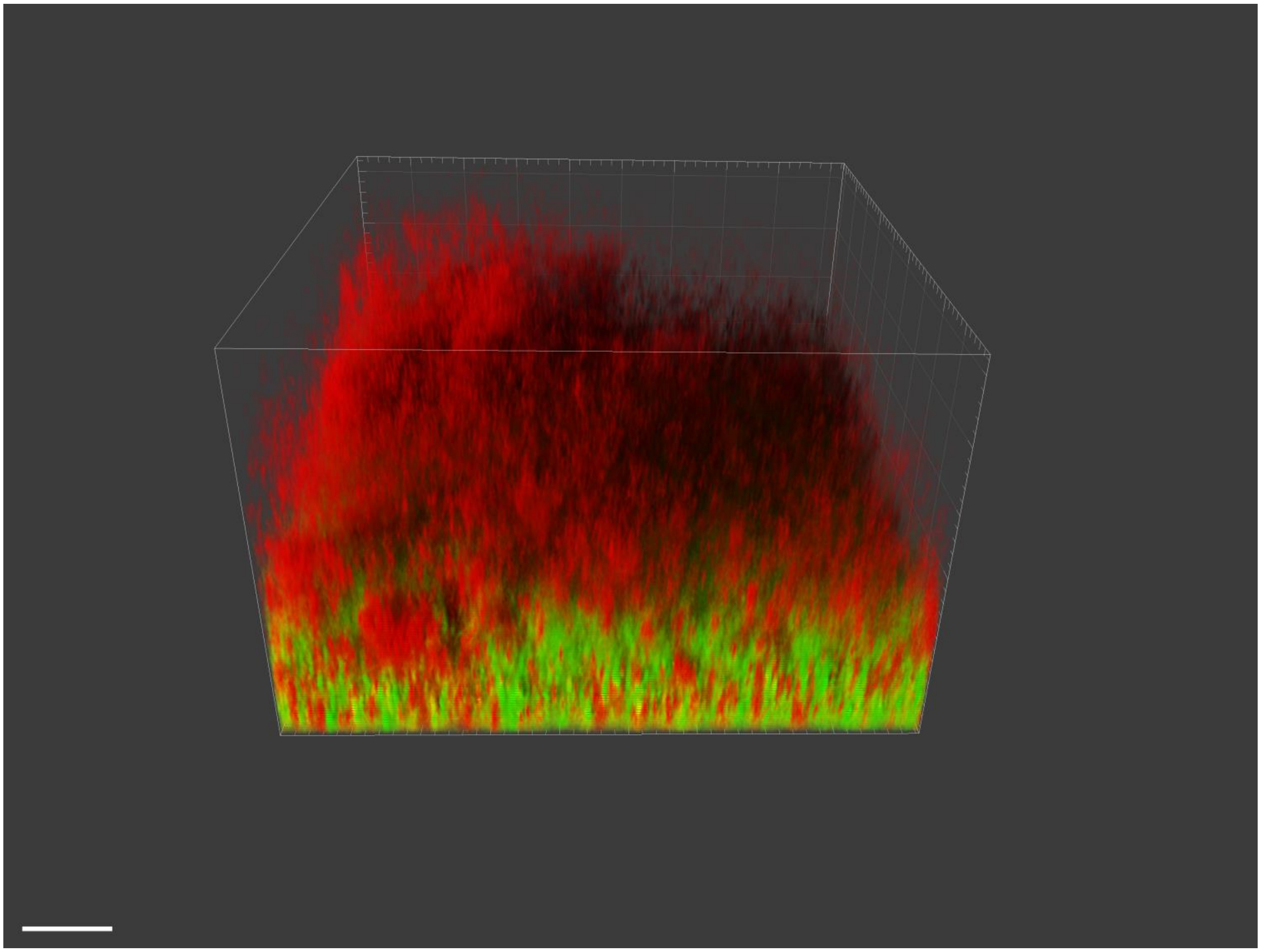

$Ab_{IC\ II}\Delta$*blp1*::p

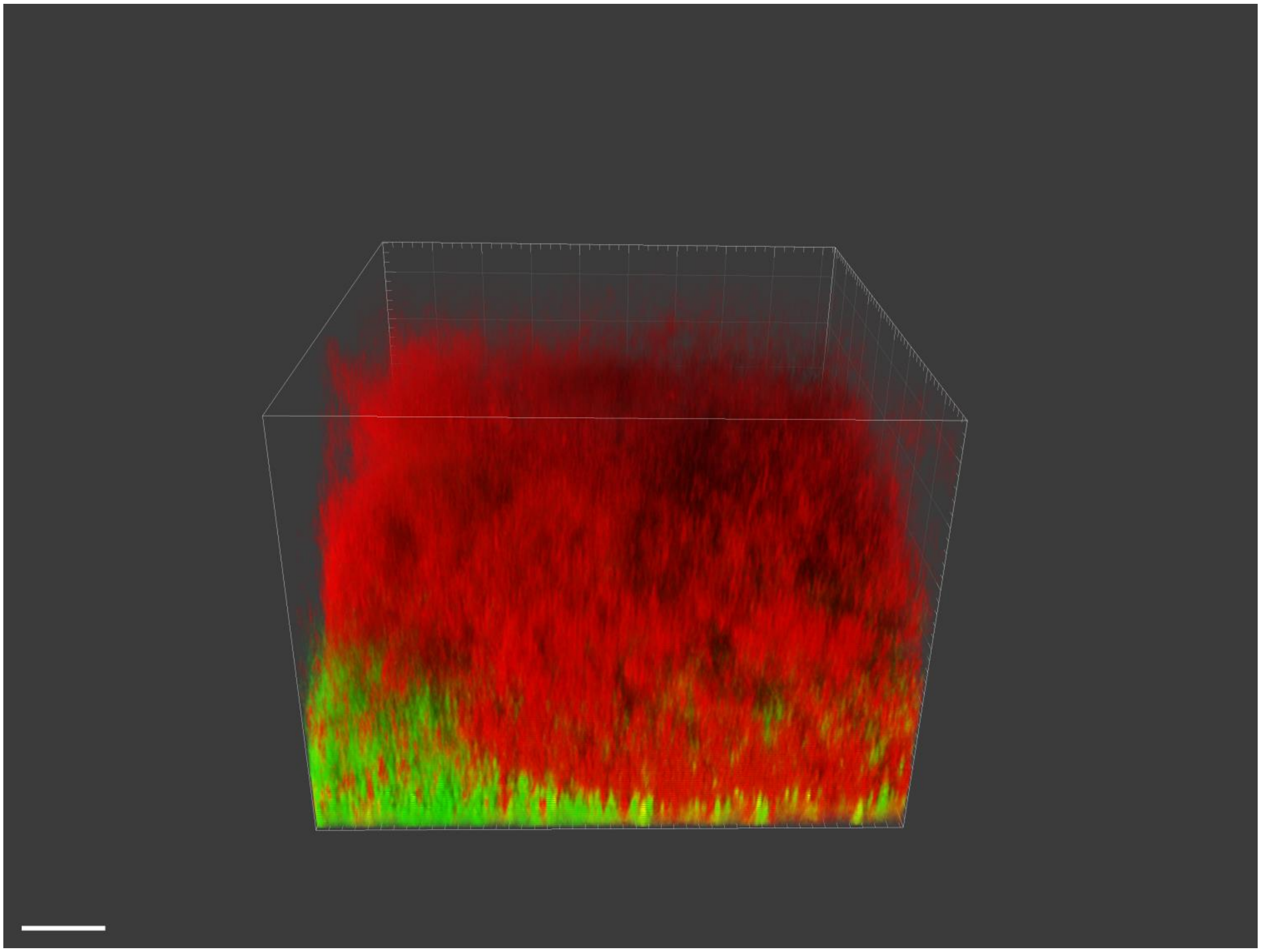

Supplemental Fig. S2. CLSM analysis of biofilms formed by the *A. baumannii* strains after 2 and 24 hours of incubation. Biofilms were stained with SYTO9 and propodeum iodide.
