# Supplementary material for: Blp1 protein shows virulence-associated features and elicits protective immunity to Acinetobacter baumannii infection
Source: BMC Microbiol. 2019 Nov 21;19:259. doi: 10.1186/s12866-019-1615-3 (PMC6873735; doi:10.1186/s12866-019-1615-3)
Supplement: Supplementary file 4 — Additional file 4: Figure S3. Expression and purification of the recombinant His-Blp12652–3362 C-terminal fragment. [file 12866_2019_1615_MOESM4_ESM.pdf]

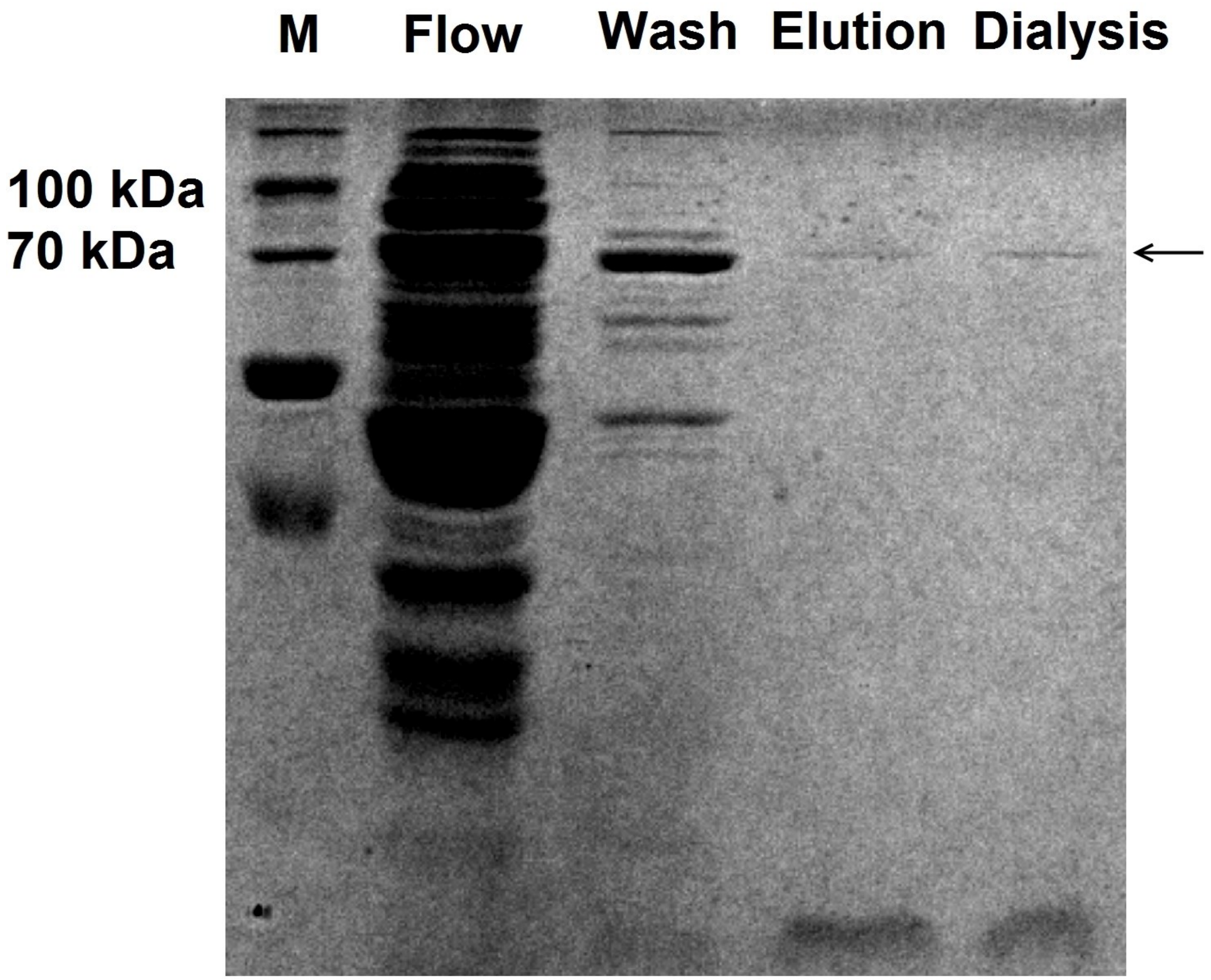

Supplemental Fig. S3. Expression and purification of the recombinant His-Blp1$_{2652\text{-}3362}$ C-terminal fragment. Fractions of flow-through, column washing, elution and dialysis in PBS buffer were fractionated in 12% SDS-PAGE. The position of His-Blp1$_{2652\text{-}3362}$ C-terminal fragment is indicated by the arrow. The positions of standard molecular mass markers (PageRuler™ Prestained Protein Ladder (Thermo Fisher Scientific)) are shown on the left.
